# Exploring target selectivity in designing and identifying PI3Kα inhibitors for triple negative breast cancer with fragment-based and bioisosteric replacement approach

**DOI:** 10.1038/s41598-024-83030-1

**Published:** 2025-01-13

**Authors:** Debojyoti Halder, Shreya Mukherjee, R. S. Jeyaprakash

**Affiliations:** Department of Pharmaceutical Chemistry, Manipal College of Pharmaceutical Sciences, Manipal Academy of Higher Education, Manipal, 576104 Karnataka India

**Keywords:** TNBC, Docking, Dynamics, Metastasis, PI3Kα, DFT, Cancer, Computational biology and bioinformatics, Drug discovery, Oncology

## Abstract

Triple-negative breast cancer (TNBC) is one of the most fatal malignancies in the world, accounting for 42% of all deaths due to metastasis. The significant development is hindered by the multi-drug resistance and poor patient compliance. PIK3CA gene mutation is one of the important causes of TNBC, which causes dysregulation of the cell cycle and cell proliferation. PI3Kα selective inhibition can decrease the TNBC by a significant level with minimal off-target effects. Novel compounds with high selectivity towards PI3Kα are crucial for treating TNBC. After extensive literature analysis, it was observed that fragment-based drug discovery, combined with structure-based virtual screening and bioisosteric replacement strategy, could provide a novel way for hit-to-lead optimization. The present study focussed on the fragment-based direct linking of 11269 moieties of the ChemDiv fragment library, - to generate novel moieties and further screened them using molecular docking, MMGBSA, and target selectivity analysis. Further, the top 2 moieties – Djh1 and Djh2 were selected after MMGBSA analysis and target selectivity prediction towards kinase. Further induced fit docking (IFD) analysis, DFT analysis, and MD simulation were employed to establish that – Djh1 and Djh2 could act as potential hit molecules for selective inhibition of PI3Kα. Further bioisosteric replacement, docking analysis, and target selectivity analysis were performed with the bioisosteres. The top two bioisosteres of Djh1 – Compound 10, Compound 06 represented excellent efficacy and selectivity towards PI3Kα in the treatment of TNBC after analysis of ADMET analysis. Further, in vitro and in vivo analysis might prove the effectiveness of the hit compounds.

**Supplementary Information:**

The online version contains supplementary material available at 10.1038/s41598-024-83030-1.

## Introduction

In the computational modeling of drug design, the utilization of fragment-based methodology created an avenue for novel molecules to be established as lead molecules. The utilization of artificial intelligence amalgamated with virtual combinatorial screening is one of the contemporary methodologies that provide an efficient, cost-effective, and less time-consuming approach to preclinical drug discovery. The process of discovery of selective molecules has gained a pace, and it has been observed that in silico drug design with target selective analysis aided a lot in the contemporary process^1,2^.

**Fig. 1:**
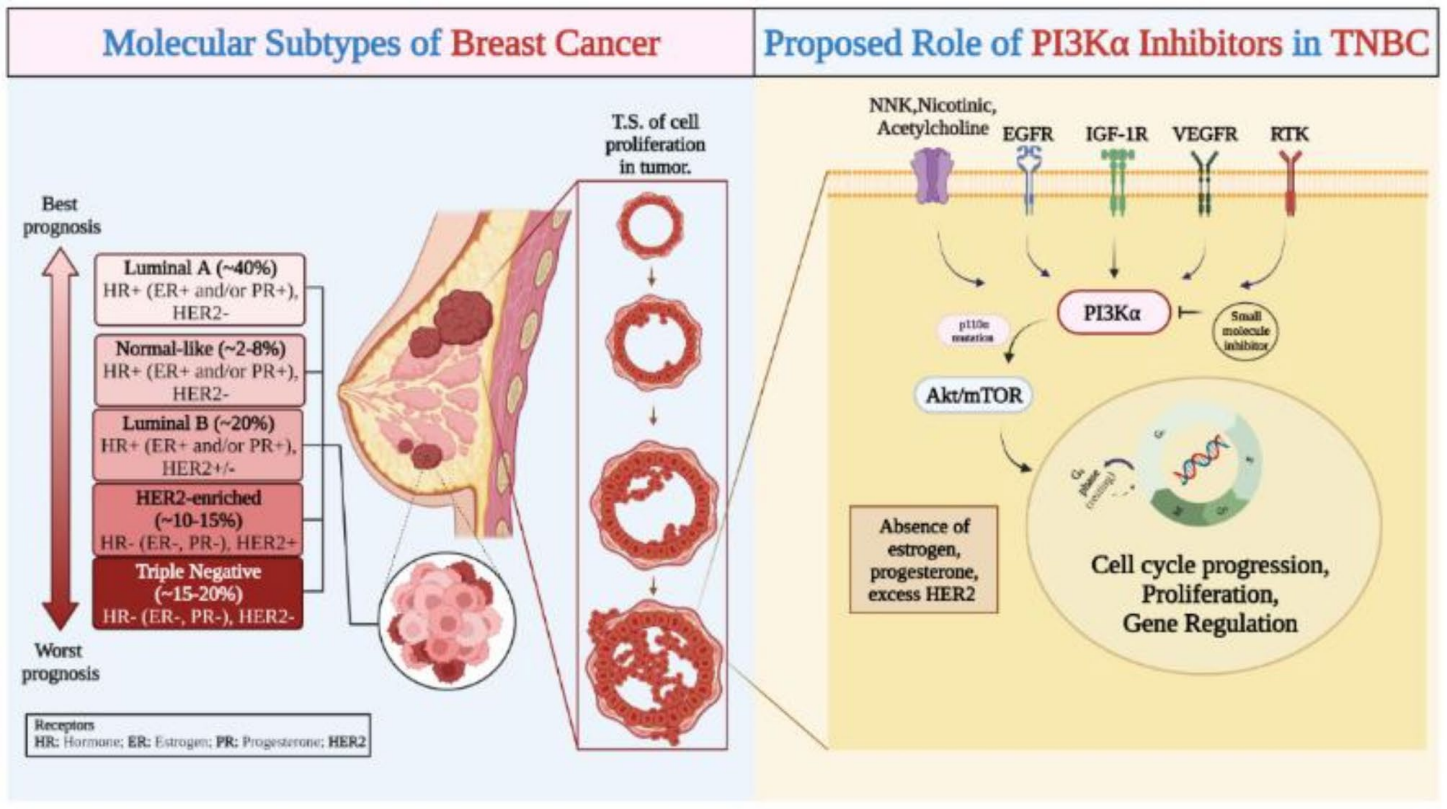
Molecular mechanism with biomarker PI3Kα in the pathophysiology of TNBC.

Triple-negative breast cancer (TNBC) is one of the most aggressive of all breast cancers, estimated to account for around 10–20% globally. Since it is very much prone to metastasis, it may remain dormant at the time of treatment but may readily come back after treatment^3^. Due to the lack of three receptors- estrogen receptor (ER), progesterone receptor (PR), and excess human epidermal growth factor receptor (HER2) from the cells, it is termed TNBC. The prognosis of the disease is the worst, and the probability of recovery is negligible^4^. Furthermore, after studying TNBC at a cellular level, the pathogenesis of the disease includes several genetic mutations like BRCA, PTEN, PIK3CA, etc., in which the prominent biomarkers for the progression of the disease are EGFR, RTK, IGF-1R, along with nicotinic and acetylcholine receptors^3,4^. After a literature survey, it was found that PI3K is one of the major targets for the regulation of growth, proliferation, and survival of cells, although the mutation of the PIK3CA gene of the p110α isoform may lead to dysregulation of cell apoptosis^5^. Therefore the possible molecular mechanism and pathophysiology of TNBC with PI3Kα pathway was reported in Fig. 1^1,6^.

Hence, the dysfunction of PI3Kα can cause TNBC, as it has been identified as a promising pharmacological research target. The primary need for novel molecule therapeutics in TNBC is due to multi-drug resistance, poor patient compliance, and off-target effects after chemotherapy. Therefore, a research gap was identified in that there is no study with the fragment-based drug design and bioisosteric replacement protocol for selective PI3Kα inhibition in the treatment of TNBC. Further, in the study of proteins related to the growth factors in TNBC,—it was well understood that prominent interactions of the ligand with amino acid residue Valine 851 for the specific alpha (α) selectivity might provide inhibitory efficacy against PI3Kα activation in TNBC^7^. Hence, the design and identification of small-molecule inhibitors could be a possible way to overcome the problem of drug resistance by combining fragment-based and structure-based protocols with the bioisosteric replacement strategy for hit-to-lead optimization.

## Materials and methodology

**Fig. 2:**
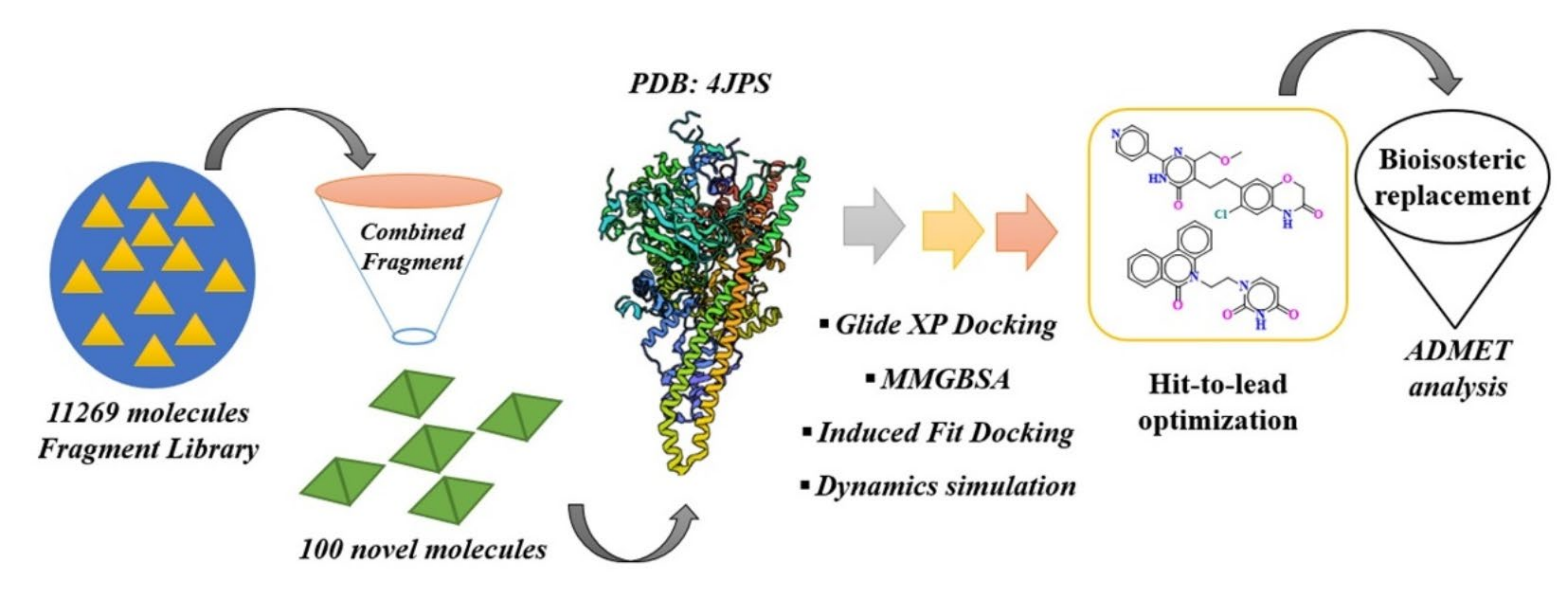
Schematic representation of the workflow.

The study was employed in a desktop equipped with INTEL CORE I5 Processor with NVidia GPU in UBUNTU OS. It was equipped with the Maestro interface of the Schrodinger suite. The toxicity analysis was performed using pkCSM open server (https://biosig.lab.uq.edu.au/pkcsm/). Figure 2 represents the schematic representation of the study workflow.

### Fragment library design

For the fragment-based design of a library, a fragment library containing 11,269 compounds was downloaded from Chemdiv, and the optimization of the ligands was executed with the LigPrep module utilizing the Epik tool at 7.4 pH, using force field OPLS4 and generation of tautomers at 1. Following the ligand preparation, the Combi Glide protocol, which is a Maestro module, was used for the enumeration of molecules and hit-to-lead optimization. The combined fragments panel can be utilized to join the fragments, where the fragments are already pre-positioned to each other. The joining method can be of two types: "Direct Joining," where the fragments will be directly joined to each other, and "Core Linking," where the fragments can be joined to core-containing molecules. Bonds that are formed between the fragments, or fragments and core-containing molecules, can be identified^8^. The identification criteria include:

- To ensure proper rotational alignment of the fragments, the angle between the bonds must be less than 15°, which is the threshold.
- To ensure proper translational alignment of fragments, the distance between atoms remaining in one fragment and leaving the other must be less than 1 Å.

Advanced Options Dialog Box is used to set the threshold values. The maximum output structures required can be specified. The fragments may overlap, and breaking an internal or peripheral bond (Hydrogen or halogen) to generate a bond with another fragment can be explored. On the other hand, the fragments are not connected if the distance between their centroids is less than a certain threshold; instead, they are thought to occupy the same spot within the Receptor. Direct linking of fragments is performed in multiple rounds^9^. The first round involves joining the pairs of fragments. The results of the previous round are used in the second round as inputs; thus, the results can combine up to four fragments and continue in a similar fashion. If the fragments lack atoms that are positioned close enough to form new bonds but are not that far apart, the system includes methylene linkers. This helps to detect if there is any possibility of the formation of a new bond. At most, two methylene linkers can join two fragments^6,10^.

In the case of core linking, methylene linkers are not involved. The fragments are not joined to each other but rather joined to a core molecule. The maximum (default 4) and minimum (default 2) number of fragments attached to individual cores can be set. The fragments are linked with the core molecule with terminal bonds like C-H and C-Halogen. Fragments will be randomly sampled in a particular number of trails (default 20). After the combination of fragments, minimization of the structures takes place^9^.

### Selection and energy optimization of receptor

The crystal structure of the kinase protein PI3Kα was selected and downloaded from PDB with code of accession 4JPS with a resolution of 2.20 Å and observed R-value of 0.206, both in acceptable ranges. Selection of any protein from UniProt requires several filters, and the selected structure meets all the requirements along with the most important—the organism of protein must be *Homo sapiens*. The X-ray crystal structure has two macromolecular structures. One is phosphatidylinositol-4, 5-bisphosphate 3-kinase catalytic subunit α, having a single chain A of 1074 sequence length and two mutations (EC: 2.7.1.153, 2.7.11.1) in PIK3CA gene. The second structure includes α regulatory subunit of phosphatidylinositol-3-kinase with single chain B of 293 sequence length and devoid of mutations. NVP-BYL719 linked N^+^H with the amino acid Valine 851, which is the unique co-crystal ligand^7^. The protein was imported into Maestro’s workspace, and the energy minimization process was performed using the Protein Preparation protocol. This is a crucial step in providing the most stable structural conformation of protein under the influence of the OPLS4 force field so that all the net atomic forces become negligible. The protein was preprocessed by filling in the missing loops and chains with the help of the Prime module at pH 7.4 ± 0. The refining process was implemented by deleting the protein’s regulatory subunit. Optimization of protein was done using PROPKA pH 7.4 water molecules beyond 3 Å were removed, and OPLS4 force field was used for minimization^11,12^.

### Molecular docking, MMGBSA, and hit analysis using target selectivity prediction

Receptor grid generation is required to represent the active site of the protein crystal structure with the help of the Glide module. The grid is generated by picking up an atom of the co-crystallized ligand. As a result, the ligand is removed from the binding site and will not take part in further ligand-receptor docking. Rotatable bonds were constrained, the Van der Waals radius was scaled by a scaling factor of 1.0, and partial charges were excluded. The validation of the docking protocol was performed in the earlier study^1^. The glide module provides the interface for performing ligand-receptor docking. Molecular docking is important for analyzing the binding pattern and efficiency between ligands and receptors. XP (Extra precision) mode was selected, and the docking score of the 100 molecules was generated from the combined fragment protocol. Glide XP docking of the standard drug Inavolisib was compared with the obtained results^13^. The ligand–protein interactions were analyzed, and the types of interactions observed were reported.

Following molecular docking analysis, MMGBSA (Molecular mechanics generalized Born surface area) calculation of the top 10 compounds was required to determine and analyze the ligand-free energy binding affinities. ΔG was calculated using the Prime module based on the theory of Generalized Born surface area (GBSA) approximation^14^. The calculation was performed for the top compounds by keeping the solvation model VSGBA 2.0 and force field OPLS4. The equation for calculation includes:

$$\begin{aligned} & \Delta G \left( { binding\;affinity} \right) \\ & \quad = \Delta G \left( {solvation\;energy} \right) + \Delta G\left( {minimized\;energy} \right) \\ & \quad + \Delta G\left( {surface\;area\;energies} \right) \\ \end{aligned}$$

The ΔG (solvation energy) is the difference between the solvation energy of the GBSA of the PI3Kα-inhibitor complex and the sum of the solvation energies for unliganded PI3Kα and the inhibitor. The ΔE (minimized energy) represents the difference between the energy of the PI3Kα-inhibitor complex and the sum of the energies for unliganded PI3Kα and the inhibitor. The ΔG (surface area energies) is the difference between the surface area energy of the PI3Kα-inhibitor complex and the sum of the energies for unliganded PI3Kα and the respective inhibitor.

The MM-GBSA Prime module determined the binding affinities and energy of optimized free receptors, free ligands, and ligand–protein complexes. The VSGB2.0 suit was used to automatically develop a solution for estimating the ligand’s strain energy, which may be displayed using Prime’s energy visualizer^14^.

### Induced fit docking, FMO analysis, and molecular dynamics simulation

Induced fit docking (IFD) follows the principle that the protein is flexible. The binding site can alter its conformation to provide a larger and more efficient binding pocket for the compounds. After Glide XP docking and MMGBSA calculation, the best molecules were chosen for IFD. In IFD, a maximum of 20 poses can be generated for a Van der Waals scaling factor of 0.50 and Prime refinement within 5 Å (standard protocol). Glide was used for docking, and the Prime module for refinement in binding poses^15^. The IFD dock scores were calculated, and the best poses were chosen for MD simulation.

The calculation for the FMO analysis (Quantum chemical descriptor analysis), single point energy (SPE), and dipole moment was calculated according to the protocol of earlier studies^1,16^.

In Molecular Dynamics (MD) simulation, Desmond provides a pharmacological environment for the compounds to bind to the catalytic binding site of protein for inhibitory activity towards PI3Kα. The three-step procedure was utilized – system building, minimization, and simulation. The system was built with a simple point charge (SPC) solvent model in an orthorhombic box of 10 Å. The system builder was used to maintain the optimum pH, neutralize the system with ions, and build the system under the OPLS4 force field. Further minimization was performed using Desmond’s Minimization tool. MD simulation was run for 100 ns, resulting in the generation of 1000 frames. The temperature and pressure were kept standard at 300 K and 1.01325 bar, respectively. The recording interval is 100 ps, and the energy is 1.2 at NPT. After completion, the simulation interaction diagram (SID) tool was used to visualize the generated reports^17^.

### Bioisosteric replacement of hit molecules

A bioisosteric replacement tool was required to replace the functional groups of the molecules to optimize their physicochemical and biological properties. The top two compounds were taken, and a bioisosteric replacement tool was used to generate novel bioisosteric structures. The set of structures was analyzed for docking interactions with PI3Kα and ADMET properties. The docking scores obtained after XP docking of the initial compounds were analyzed. The ligand-receptor interactions were reported along with the target selectivity analysis^18^.

### ADMET comparison

ADMET prediction is required to analyze the drug-likeness of the molecules and the standard drug used, i.e., Inavolisib. The QikProp module in Maestro and pkCSM of Biosig Lab were used to predict drug-likeness and ADMET properties. The criteria for determination of the drug-likeness is Lipinski’s rule of five. Properties like H-bond donor, H-bond acceptor, predicted octanol/water partition coefficient (logPo/w), GI absorption, BBB permeation, hERG I and II inhibition (for toxicity analysis), total clearance, and other such properties were analyzed. The properties of the top two compounds and their bioisosteres, along with Inavolisib, were reported and analyzed^19,20^.

## Results and discussions

One of the most important biological targets for anticancer drug discovery, especially for the discovery of inhibitors targeting PI3Kα, and the combination of fragment-based virtual screening for novel hit optimization is considered an excellent methodology for its time efficiency- and eradication of false negatives in the computer-aided drug design and screening in the treatment of TNBC. The selection of the target receptor directly relates to the success rate of the fragment-based protocol. The protein selection (PDB ID: 4JPS) with catalytic binding pocket relates with the identification with relevant co-ligand,—the X-ray crystal structure of the Receptor with a resolution of 2.2 Å of *Homo sapiens *organism, with mutations of PIK3CA gene at the primary chain A, signifies the protein is druggable for selective PI3Kα inhibitors. The selective PI3Kα inhibition can be performed as it has a druggable catalytic binding site with amino acid residue Valine 851, in which PI3Kα selective inhibitor Alpelisib (NVP-BYL719) is present. The UniProt KB database aided in the selection of 4JPS from 89 proteins of the P42336 repository, as reported in earlier studies, as well as the mutation present in the protein^18,21^. The model of the rationalization of the contemporary study is a quest for superior selectivity and potency along with the reduction of off-target effects and the inhibition of mutation. Further, the optimization of 4JPS with the Protein Preparation protocol on the basis of local energy minimization by preprocess, refining, and minimization at a pH of 7.4 by prefilling the side chains and loops using the Prime module, and the force field for minimization was assigned as OPLS4. The fragment library of 11,269 moieties was imported from the ChemDiv database and optimized using the LigPrep module for the generation of 3D conformer and, further, the novel molecule development using the Combine Fragment module, which is based on the principle of Combi Glide,—a tool for high-throughput virtual screening. The combined fragments protocol provides us with the 100 molecules by random screening and the bond-forming ability of the fragments. Furthermore, the virtual screening by protein–ligand docking analysis with PI3Kα makes the process possible for the selection of the top ten compounds. The binding interaction and MMGBSA energy state were calculated using the Glide and Prime module. The target selectivity prediction was explored to understand and validate the study utilizing the open server of Swiss Target Prediction for the selective PI3Kα inhibition in the treatment of TNBC^22^. The current study explored the relationship between fragment-based molecule developments, their receptor binding analysis, and target selectivity prediction of the designed molecules from combining fragments.

In our earlier studies, the docking protocol for the PDB ID: 4JPS was already validated^1^, and hence, the generated novel molecules were utilized with receptor-ligand docking analysis, which led to the selection of the top ten molecules from the novel molecules developed – Djh1 to Djh10, and the data was compared with the selective PI3Kα inhibitor Inavolisib.

### Receptor-ligand docking, binding energy analysis, and fragment analysis

**Fig. 3:**
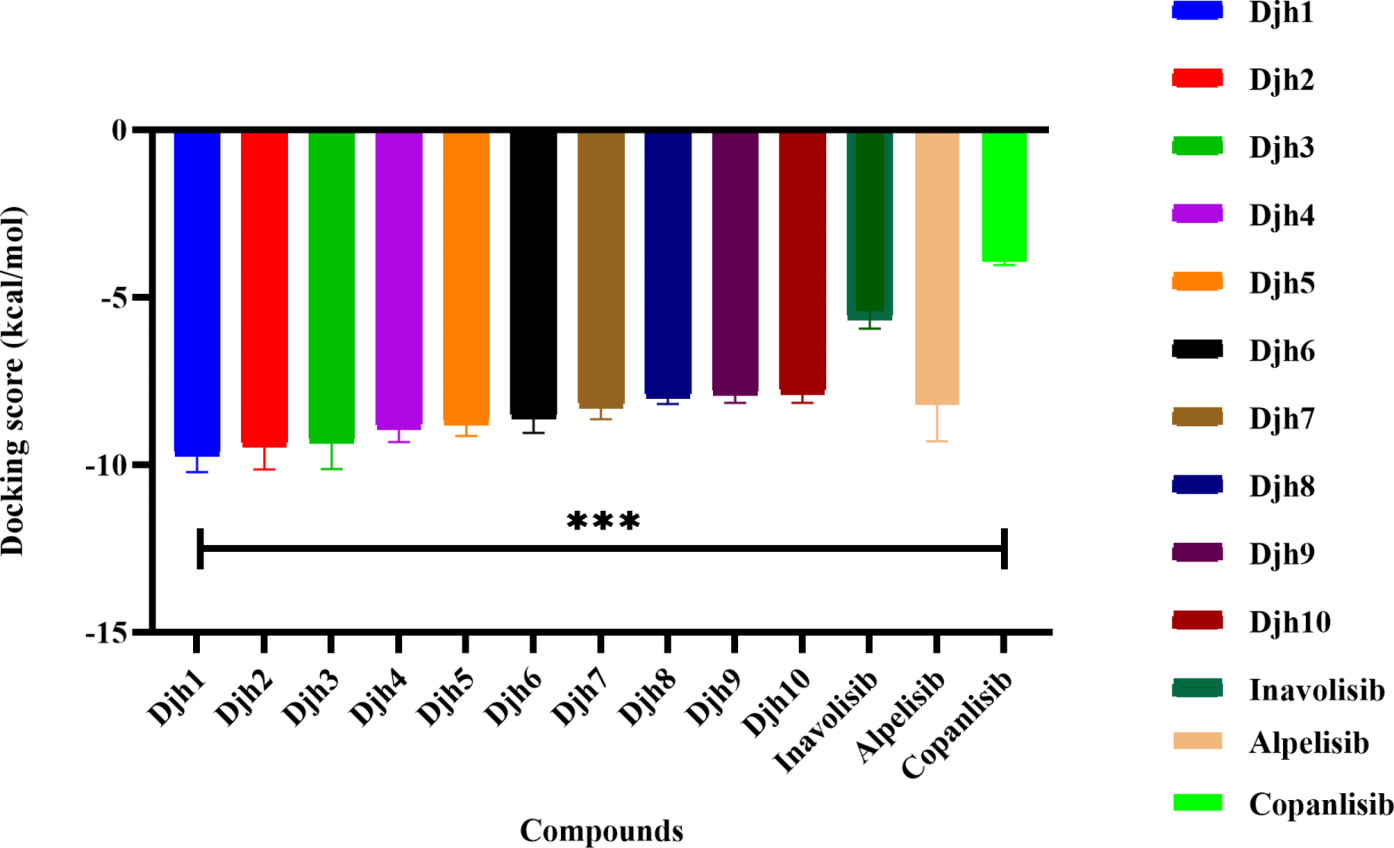
The graphical representation of the analysis of docking scores of Djh1 to Djh10, with *n* = 2, r2 = 0.9004 (*p* < 0.0001) by One-way ANOVA, the study was significant, comparing with the controls [PI3Kα inhibitors (Inavolisib, Alpelisib, and Copanlisib)].

The study of receptor-ligand binding analysis at the catalytic binding region is a framework for how a drug molecule binds the receptor at its active site to inhibit the protein. This leads to the identification and optimization of new leads upon understanding the underlying pharmacological mechanisms and chemistry. The non-covalent interactions, such as hydrogen bond interaction, stacking of π-π bond, etc., played various significant roles in the framework of ligand-reception binding. The current study focussed on the inhibition of PI3Kα mutation of the PIK3CA gene, which specifically leads to uncontrolled cell growth and loss of cellular apoptosis. The identification of a specific amino acid for the inhibition of PIK3CA mutation is Valine851 residue, and the Hydrogen bond interaction with the chemical moiety can induce apoptosis and downregulate the uncontrolled growth and proliferation. After the docking of 100 novel compounds, generated from the fragment linking towards the protein catalytic binding site, the best ten compounds – Djh1 to Djh10, along with their structure, IUPAC names, docking score in kcal/mol, MMGBSA binding free energy score, and other binding properties – MMGBSA (ΔG Coulomb), and SASA (Å^2^) at the catalytic binding site of PI3Kα, reported in Table 1, and IUPAC name was provided in Supplementary Table S1 (Fig. 3).

**Table 1:** The top ten compounds – Djh1 to Djh10 with their structure, docking score (kcal/mol), MM-GBSA ΔG (kcal/mol), MMGBSA (ΔG-Coulomb), and SASA (Å^2^).

Compound ID	Structure	XP docking score (Kcal/mol)	MM-GBSA ΔG (Kcal/mol)	MM-GBSA (ΔG-Coulomb)	SASA (Ǻ^2^)
Djh1		−10.214	−60.70	−12.92	478.168
Djh2		−10.126	−45.64	−5.60	408.104
Djh3		−10.122	−43.20	−12.97	436.787
Djh4		−9.308	−58.36	−16.25	354.017
Djh5		−9.127	−54.32	−15.86	377.260
Djh6		−9.040	−63.18	−5.02	451.660
Djh7		−8.630	−63.04	−11.42	354.017
Djh8		−8.172	−67.98	4.61	446.080
Djh9		−8.144	−60.71	−19.57	393.844
Djh10		−8.139	−64.20	−4.54	354.017
Inavolisib		−5.922	−57.51	−20.54	635.095
Alpelisib		−9.295	−50.27	−12.78	711.630
Copanlisib		−4.029	−21.56	−3.55	-

The docking protocol follows the structure-based screening of the molecules, using the Glide Extra precision module after the grid generation, which basically calculates the binding site by deleting the co-ligand and making it suitable for other minimized ligands to bind at the specific binding pocket. The virtual screening of 100 molecules was performed, and the top ten molecules were selected according to the docking score and further with the MMGBSA scores. The docking score, MMGBSA scores, and SASA, top ten molecules were compared with Inavolisib, reported in Table 1.

**Fig. 4:**
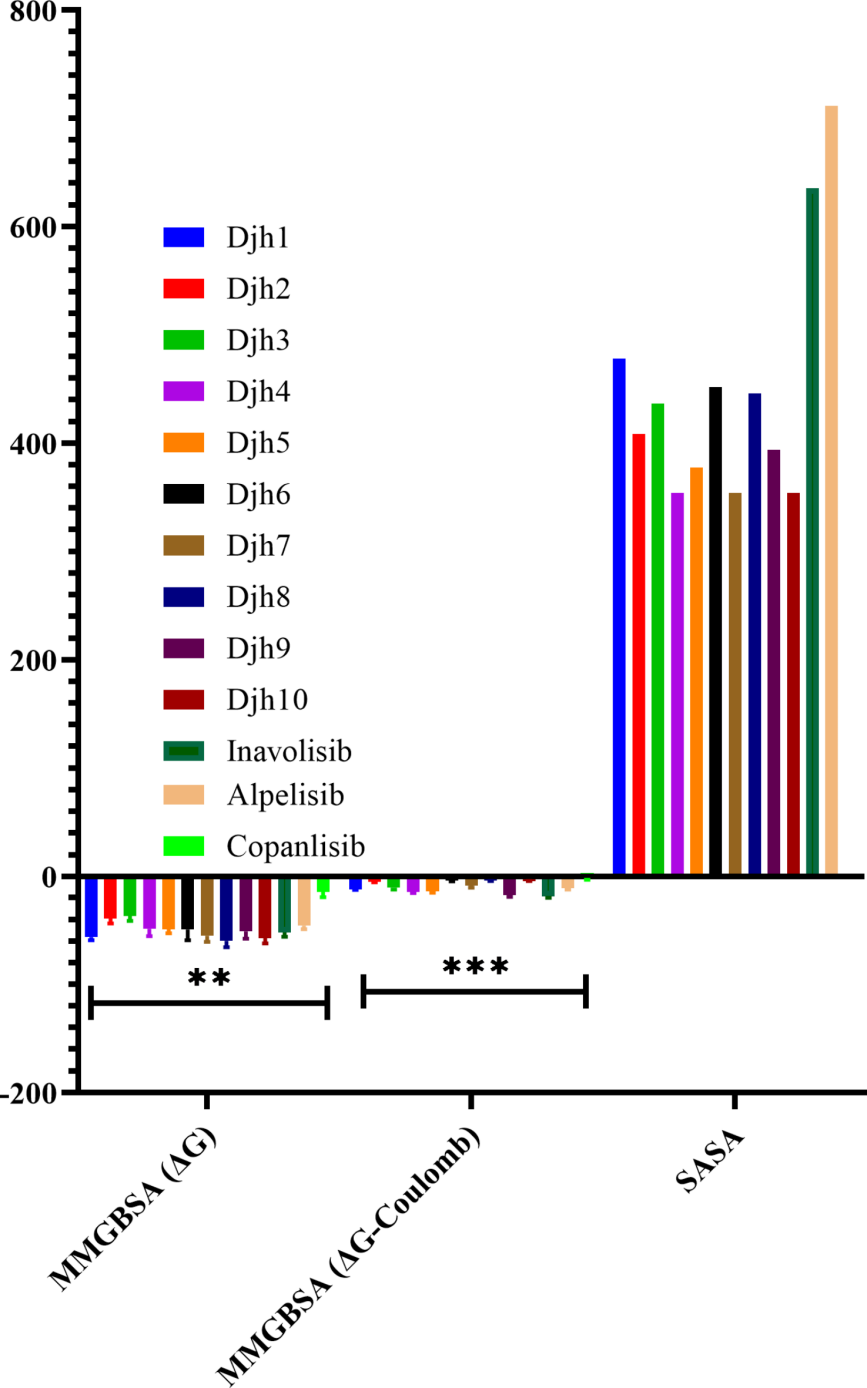
Graphical representation of the MMGBSA (ΔG) [*n* = 2, *p* < 0.05 (significant), r^2^ = 0.8127 (One-way ANOVA)], MMGBSA (ΔG-Coulomb) [n = 2, *p* < 0.0001 (significant), r^2^ = 0.9507 (One-way ANOVA)], and SASA of the top ten compounds – Djh1 to Djh10.

From the analysis of Table 1, it was observed that the top ten molecules generated from the combined fragment protocol – Djh1 to Djh10 represented excellent docking score in the range of −10.214 to −8.139 kcal/mol and MMGBSA ΔG score of −67.98 to −43.20 kcal/mol. While comparing with the docking score of Inavolisib (−5.922 kcal/mol), the top ten molecules represented a much superior outcome, and the MMGBSA ΔG score is −57.51 kcal/mol, which is in the acceptable and similar range to the top ten molecules. The total solvent accessible surface area (SASA) has a relevant role in the stability of ligands at a specific binding site, and it must be in the range of 300–1000 Å^2^. Hence, the top ten molecules represented ligand SASA in the range of 350–480 Å^2^, compared with Inavolisib at 635.095 Å^2^. Further, the MMGBSA ΔG Coulomb of the top ten compounds, along with Inavolisib, is in the range of −4 to −20. The graphical representation of the MMGBSA ΔG, MMGBSA ΔG-Coulomb, and SASA is provided in Fig. 4. Further for statistical analysis of docking score, and error analysis for molecular docking, redocking was performed by changing only one parameter of extra precision (XP) mode to standard precision (SP) mode, for molecules Djh1 to Djh10 along with standard selective PI3Kα inhibitor Inavolisib, Alpelisib, and PI3K pan inhibitor Copanlisib. Therefore, the docking study with error analysis, having the parameters of n = 2(duplicate), and calculated the standard error of mean (SEM) and plotted in the Graphpad prism 8.1, reported in the Supplementary Table S4, and the graphical representation was provided in the Fig. 3.

The docking score and the binding analysis of MMGBSA and SASA provide information on the relationship between ligand and protein binding affinities. From the above analysis, it was observed that Djh1 and Djh2 showed the best molecule-receptor binding score, along with an acceptable range of free binding energy analysis when compared with PI3Kα selective inhibitor Inavolisib. Further, the receptor-ligand interaction analysis, along with the target selectivity analysis, was provided in Table 2. Following the analysis of the binding energy and docking score of the top ten molecules – Djh1 to Djh10, the binding interaction analysis provides insight into the catalytic binding site and its importance in the inhibition of the PI3Kα. Among the various amino acid residues, the hydrogen bond interaction of ligand moiety with Valine851 represented PI3Kα inhibition as reported in earlier studies; similarly, we also observed that the top ten compounds – Djh1 to Djh10 showed interaction with Valine 851.

**Table 2:** The top two compounds – Djh1 and Djh2 (2D interaction diagram), were compared with Inavolisib with target selectivity analysis with Inavolisib.

Compound ID	Interaction Diagram (2D)	Important interactions	Target Selectivity
Djh1		**H-bond:** V851, Y836, **π-π stacking:** Y836, H855, W780	
Djh2		**H-bond:** V851, **π-π stacking:** W780	
Inavolisib		**H-bond:** Q859, S854, D933, **π-π stacking:** Y836	
Alpelisib		**H-bond:** V851, S854, Q859	
Copanlisib		**H-bond:** V851, N853, D933, **π-π stacking:** W780, **π-cation:** Y836	
**[Note:** A (Alanine), R (Arginine), N (Asparagine), D (Aspartic acid), C (Cysteine), E (Glutamic acid), Q (Glutamine), G (Glycine), H (Histidine), I (Isoleucine), L (Leucine), K (Lysine), M (Methionine), F (Phenylalanine), P (Proline), S (Serine), T (Threonine), W (Tryptophan), Y (Tyrosine), V (Valine)**]**			

From the analysis of Table 2, it was observed that Djh1 represented H-bond interaction with Valine851 and Tyrosine836 and stacking of π-bond with Tyrosine836, Histidine855, and Tryptophan780 amino acid residues. The compound Djh2 represented a similar H-bond interaction with Valine851 and stacking of π-bond with Tryptophan780. Further, on analysis of Djh3, Dh4, and Djh10, it was observed that all three molecules represented H-bond interaction with Valine851 and Tyrosine836. The stacking of π-bond with Tyrosine836 was observed in the Djh3 and Djh6, while Djh5 and Djh10 showed stacking of π-bond with both Tyrosine836 and Tryptophan780. The compounds – Djh4, Djh7, Djh8, and Djh9 also presented interaction with π-bond (stacking) with Tryptophan780. A few compounds, like Djh3, Djh7, Djh6, Djh8, and Djh10 showed halogen bond interactions with Aspartic acid933, Aspartic acid810, and Valine851 residues. All the top ten molecules provided hydrophobic interactions with common amino acid residues, such as Valine851, Valine850, Isoleucine848, Tyrosine836, Isoleucine932, Phenyl alanine930, Phenyl alanine934, Leucine807, Isoleucine800, Methionine922, Methionine772, Tryptophan780; positive charge interaction with Lysine802, Arginine852, negative charge interaction with Aspartic acid933, Aspartic acid810, Glutamic acid849, Glutamic acid798, and Polar interaction with Glutamine859, Threonine856, Histidine855, Serine854, and Asparagine853. The PI3Kα inhibitor Inavolisib did not show any H-bond interaction with Valine851 residue but represented interaction with Glutamine859, Serine854, and Aspartic acid933; stacking of π-bond was represented with Tyrosine836 in the same binding pocket. The top eight molecules – Djh3 to Djh10 – interaction diagram (2D), along with important interactions, was provided in Supplementary Table S2. After the analysis of binding affinities and interactions of the top ten compounds – Djh1 to Djh10 along with Inavolisib, the compounds Djh1 to Djh10 showed superior binding affinities and better interactions than selective PI3Kα inhibitor Inavolisib, and Alpelisib, as well as PI3K pan inhibitor Copanlisib^23^. All 3D interaction diagrams were reported in the Supplementary Figure S1 – Supplementary Figure S11, Supplementary Figure S26, and S27. Further, the enrichment analysis was provided in the Supplementary Figure S19.

Furthermore, the target selectivity prediction provides insight into the selectivity of the novel molecules towards the target, which is required to decrease the off-target effects as well as toxicity. As PI3Kα is an enzyme of the Kinase class, the top two moieties – Djh1 and Djh2 provided excellent kinase selectivity of 86.7% and 53.3% while compared with Inavolisib of 20% kinase selectivity, and Alpelisib of 6.7%. Hence, the importance of Valine 851 residue was clearly observed in the inhibitory binding site of the PI3Kα as the H-bond interaction is the strongest of all non-covalent interactions, the compounds – Djh1 and Djh2 with superior binding affinity showed greater docking scores and binding affinity as well as kinase selectivity. Therefore, Valine851 residue is very crucial in PI3Kα inhibitory potency and selectivity. For the analysis of the fragments of the top two compounds – Djh1 and Djh2, selected after various filters, docking score, free binding energy score, binding interaction, and target selectivity analysis with amino acids. Further analysis was carried out using only Inavolisib as positive control, as it was representating best docking score, and better kinase selectivity than Alpelisib and Copanlisib in the in silico study.

### Fragment analysis

**Fig. 5:**
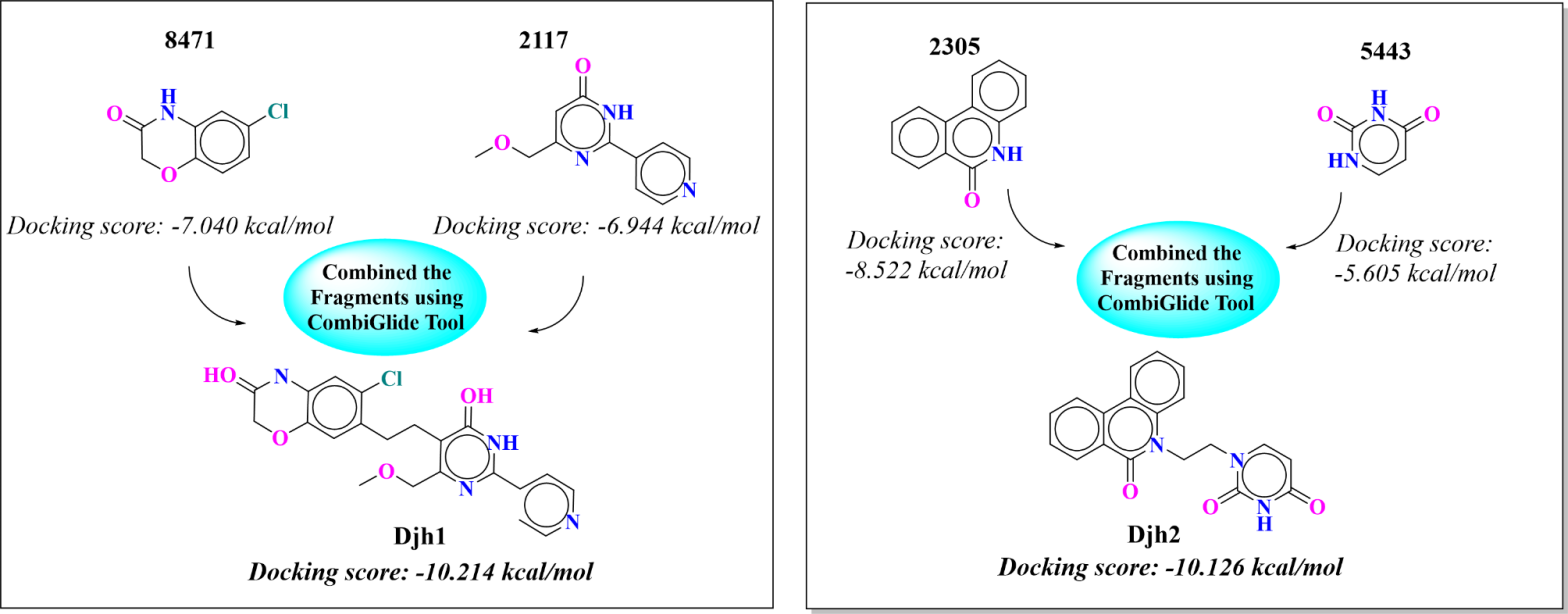
Fragment joining of the top two compounds – Djh1 and Djh2 using the ethyl bridge.

Out of the top 10 compounds, the top 8 compounds share a similar pharmacophore- 8471 [6-chloro-2H-benzo(b)(1,4)oxazin-3(4H)-one] – which is also present in the best compound – Djh1. Another fragment present in the Djh1 is 2117, which consist of [6-(methoxymethyl)−2-(pyridin-4-yl)pyrimidin-4(3H)-one] moiety. While combining both the fragments with methylene linker utilizing the Combine Fragments protocol of Maestro, the docking score of the compound increased to −10.214 kcal/mol, while the fragments – 8471 and 2117 only expressed a docking score of −7.040 kcal/mol, and −6.944 kcal/mol respectively. Similarly, the compound Djh2 consists of two fragment moiety – 2305 [phenanthridin-6(5H)-one] and 5443 [pyrimidine-2,4(1H,3H)-dione] with docking score of −8.522 kcal/mol and −5.605 kcal/mol respectively. However, when these two fragments are linked with the methylene group to form Djh2, the docking score (−10.126 kcal/mol) and binding affinity towards PI3Kα enhanced like Djh1. The analysis is reported in Fig. 5.

Hence, it was observed that the combined fragment protocol is very efficient in enhancing the molecular potency and selectivity towards a specific target, similar to our study in the quest for novel inhibitors targeting PI3Kα. Along with that, it was observed that novel lead molecules can be designed with benzo-oxazine and pyrimidine moieties for selectivity towards PI3Kα. The combination of fragment-based molecule design and structure-based computational screening provides an excellent way for hit-to-lead optimization.

### Induced fit docking analysis

After the fragment-based molecular design of 100 molecules and receptor-based screening in the PI3Kα catalytic binding pocket, the top two molecules – Djh1 and Djh2 were selected according to the docking score, MMGBSA calculations, binding interaction with the amino acid residue Valine851, as well as the target selectivity prediction. In the receptor-ligand Glide docking analysis, the Receptor remains rigid, and only the best docking pose can be observed with a limited number of outputs. Therefore, to overcome these problems, proper validation of the ligand-receptor interaction, and extensive study of the binding interaction of the ligand with the Receptor in different poses, the protocol for IFD was applied, which utilized the Glide and Prime module. The protocol runs on the Python script, which automates the structure-based screening procedure. It provides multiple binding modes of a single compound at the catalytic binding site, which helps abolish the false negatives from the ligand-receptor docking analysis. The output of the IFD score also signifies the binding affinity in the inhibitor site, i.e., the greater the IFD score, the better the possibility of a more stable and selective compound towards a specific pharmacological target.

**Fig. 6:**
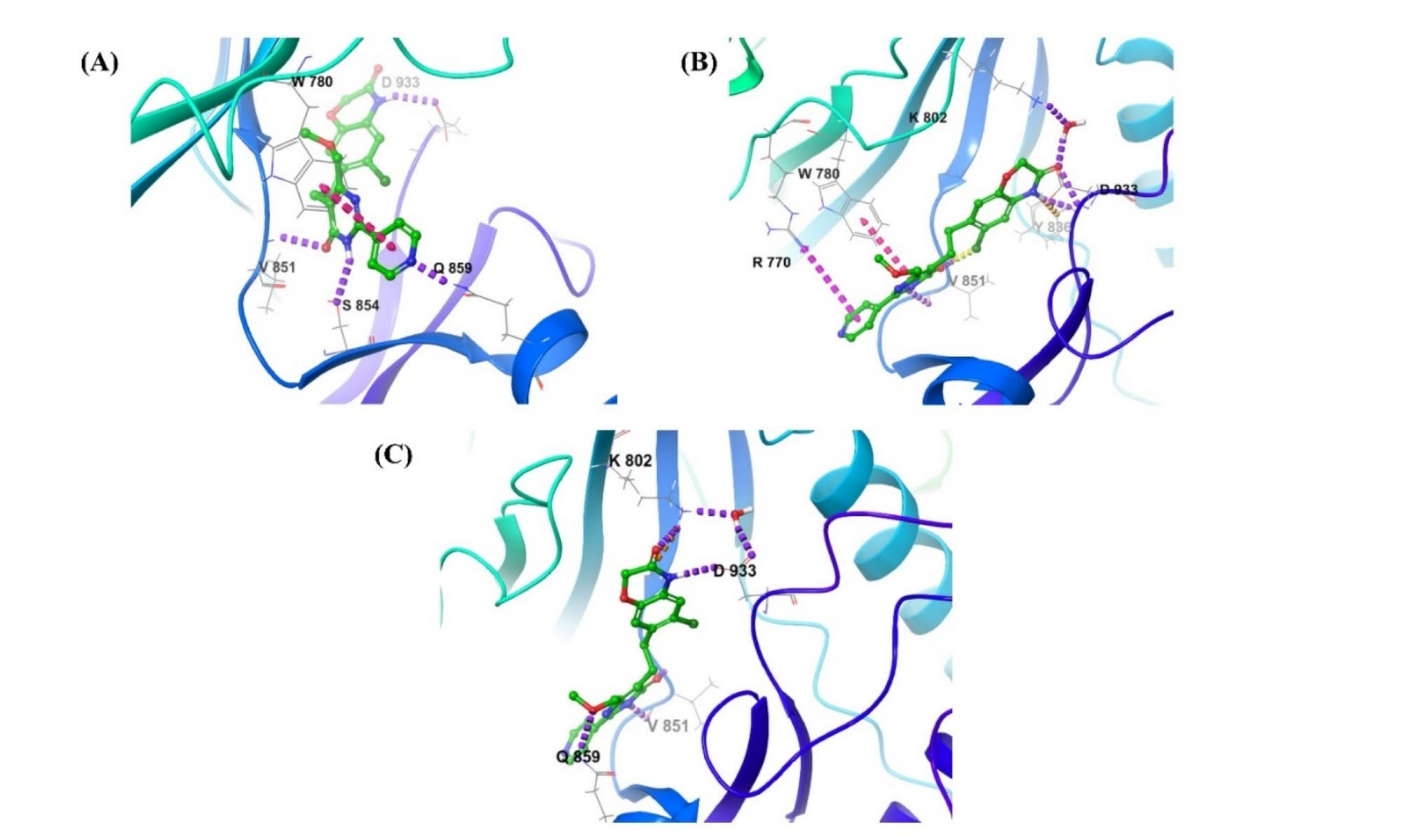
3D interaction of IFD for Djh1, with IFD scores of poses—(**A**) 2817.56, (**B**) 2817.25, and (**C**) 2816.81.

From the analysis of the Djh1 and PI3Kα docking, utilizing the induced fit mode,—ten poses were generated. The top three poses were analyzed, and it was observed that pose (A) of Djh1 has the highest IFD score of 2817.56, and the binding interaction with the amino acid residues—Valine851, Tryptophan780, Serine854, Glutamine859, Aspartic acid933 provided excelling binding affinity towards the catalytic binding region of the PI3Kα. Pose (B) also represented a similar interaction to pose (A) with Valine851, Tryptophan780, Tyrosine836, Aspartic acid933, Lysine802, and Arginine770 and the IFD score was slightly less than pose (A), which is 2817.25. The pose (C) represented the lowest IFD score among the top three poses, although it showed excellent interaction with Valine851, Glutamine859, Lysine802, and Aspartic acid933. Hence, it can be concluded that the superior interaction with Valine851 showed a better IFD score along with binding affinity towards the inhibition of PI3Kα in the treatment of TNBC, reported in Fig. 6. The 2D binding interaction was provided in the Supplementary Figure S12.

**Fig. 7:**
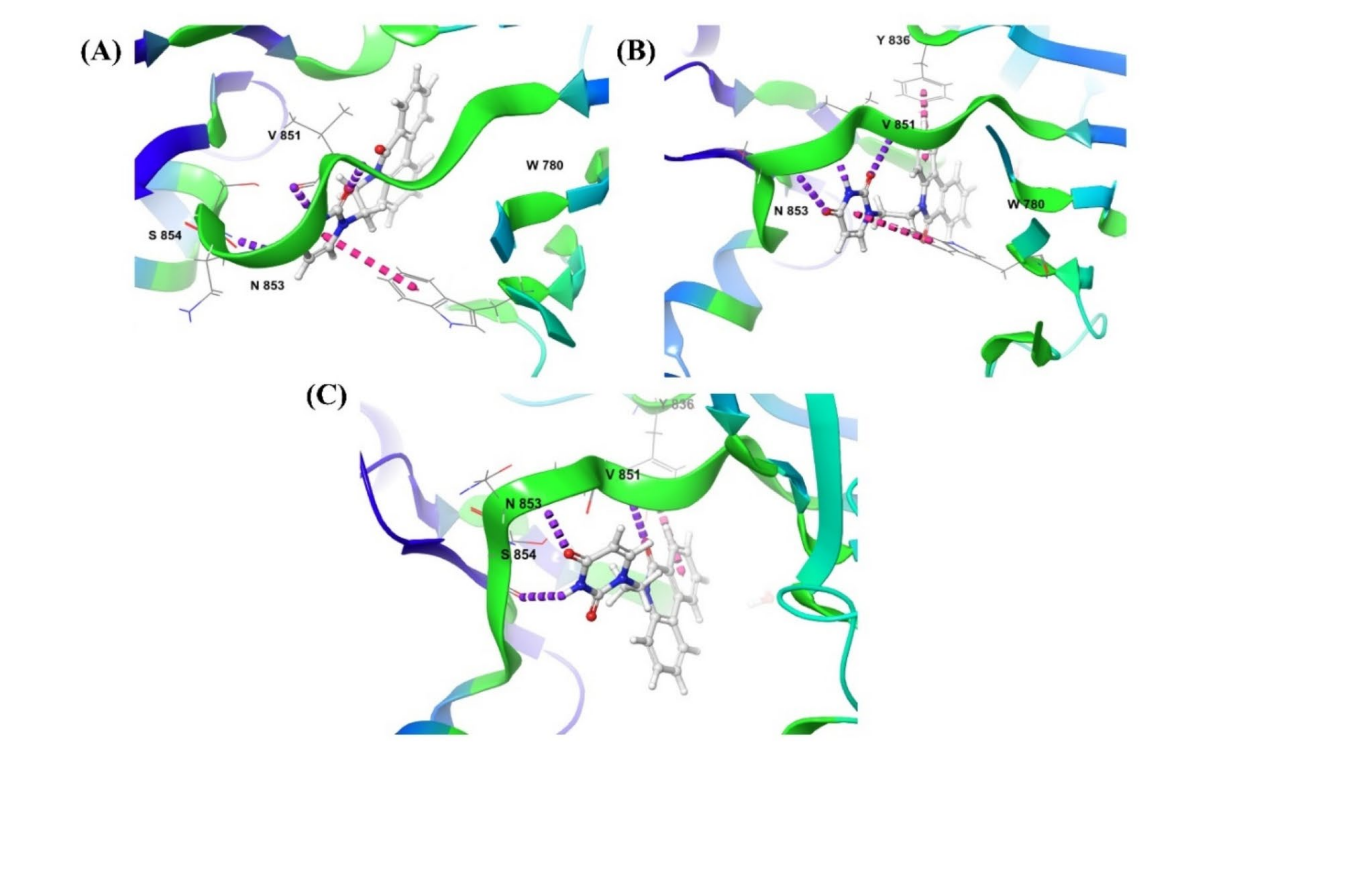
3D interaction of IFD for Djh2, with IFD scores of poses—(**A**) 2812.73, (**B**) 2811.93, (**C**) 2811.93.

The analysis of Djh2 represented the output of 10 structures with PI3Kα-Djh2 complex, out of which the top three poses were reported in Fig. 7, in which pose (A) the IFD score of the pose (A) was 2812.73, which is lesser than the top three poses of Djh1, but the H-bond interaction with Valine851, Serine854, and Asparagine853 and π-bond interaction with Tryptophan780 is very significant in the inhibitory action. Similarly, poses (B) and (C), with IFD scores of 2811.93, showed H-bond interactions with Valine851, Serine854, and Asparagine853, and π-stacking interaction with Tyrosine836, and Tryptophan780. The 2D binding interaction was provided in the Supplementary Figure S13.

**Fig. 8:**
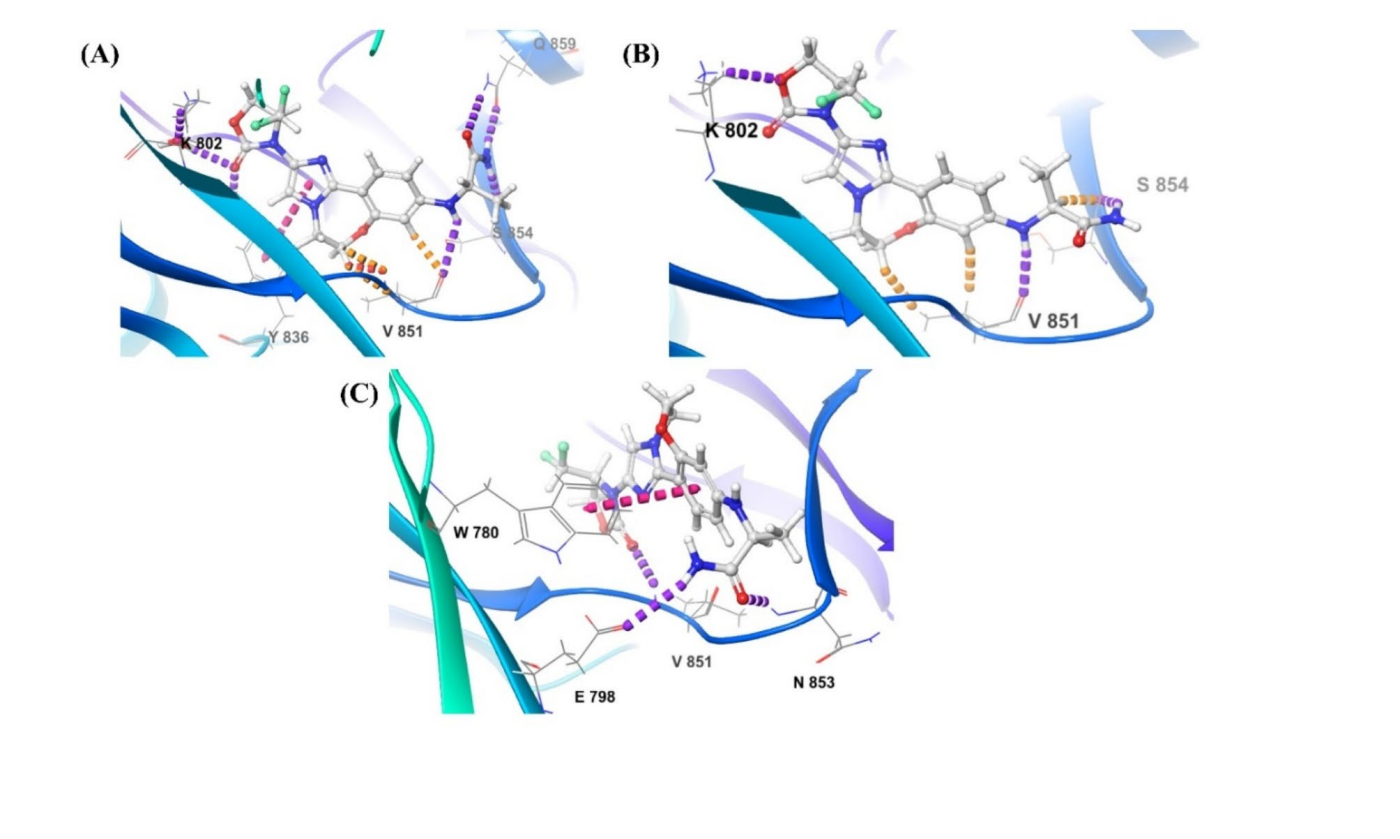
3D interaction of IFD for Inavolisib, with IFD scores of poses—(**A**) 2812.64, (**B**) 2811.82, (**C**) 2810.18.

Further, Inavolisib represented the IFD score of the top three poses – (A), (B), and (C) were 2812.64, 2811.82, and 2810.18, respectively, reported in Fig. 8. The pose (A) presented H-bond interaction with Valine851, Serine854, and Glutamine859, the pose (B) presented H-bond interaction with Valine851, and Asparagine853, and the pose (C) also presented H-bond interaction with Valine851, Serine854, and Asparagine853. And the π-bond stacking interaction was represented with Tryptophan780 and Tyrosine836. The 2D binding interaction was provided in the Supplementary Figure S14.

While comparing Djh1 and Djh2 with Inavolisib, it was observed that Djh1 showed superior interaction with Valine851 and IFD score than Djh2 and Inavolisib in all three poses, which signifies its importance in the binding site for inhibitory efficacy. Further, Djh2 showed a lower IFD score and binding affinity than Djh1, although the IFD score in pose (A) of Djh2 was better than in pose (A) of Inavolisib. Similar cases were observed in poses (B) and (C). Therefore, it can be stated that Djh1 is the best molecule for further analysis, representing the best docking score and excellent free binding energy analysis, with a greater IFD score when compared with the standard PI3Kα inhibitor Inavolisib. The compound Djh2 also presented a better binding score (docking) than the standard drug Inavolisib and a superior IFD score while comparing the top three poses with Inavolisib. Hence, the top two compounds – Djh1 and Djh2 were further analyzed utilizing the quantum chemical descriptors for the stability analysis and dynamic simulation with PI3Kα.

### Analysis of Frontier Molecular Orbital (FMO)

There are several applications of density function theory (DFT) in drug design and hit-to-lead optimization, such as the interaction analysis of ligands and receptors, modeling of the mechanism of action of drugs, and stability analysis of ligands. The top two molecules selected after fragment-based combination, receptor-ligand binding analysis, docking score, IFD score along with target selectivity prediction analysis,—Djh1 and Djh2, would be utilized for quantum chemical analysis using the (B3LYP-D3) 6-31G** theory. The analysis of FMO by calculating the HOMO and LUMO parameters using the Jaguar module of Maestro influences the stability and reactivity of molecules at the target binding site utilizing several descriptors. The potential energy of the molecule was calculated by Born–Oppenheimer approximation of *U* = *Ee* + *Vnn*, i.e., *U* represented the single point energy, *Ee* was electronic energy, and Vnn was nuclear repulsion potential energy. The SPE and dipole moment of the compounds – Djh1, Djh2, and Inavolisib were calculated and reported in Table 3.

**Table 3:** Single point energy of the top two compounds – Djh1, Djh2, and Inavolisib with dipole moment.

*Compound ID*	DFT(B3LYP-D3) 6-31G**	
	Single Point Energy [U *eV*]	Dipole moment
Djh1	- 4.8757 × 10^4^	3.187
Djh2	- 3.0561 × 10^4^	4.363
Inavolisib	- 4.048 × 10^4^	7.564

**Fig. 9:**
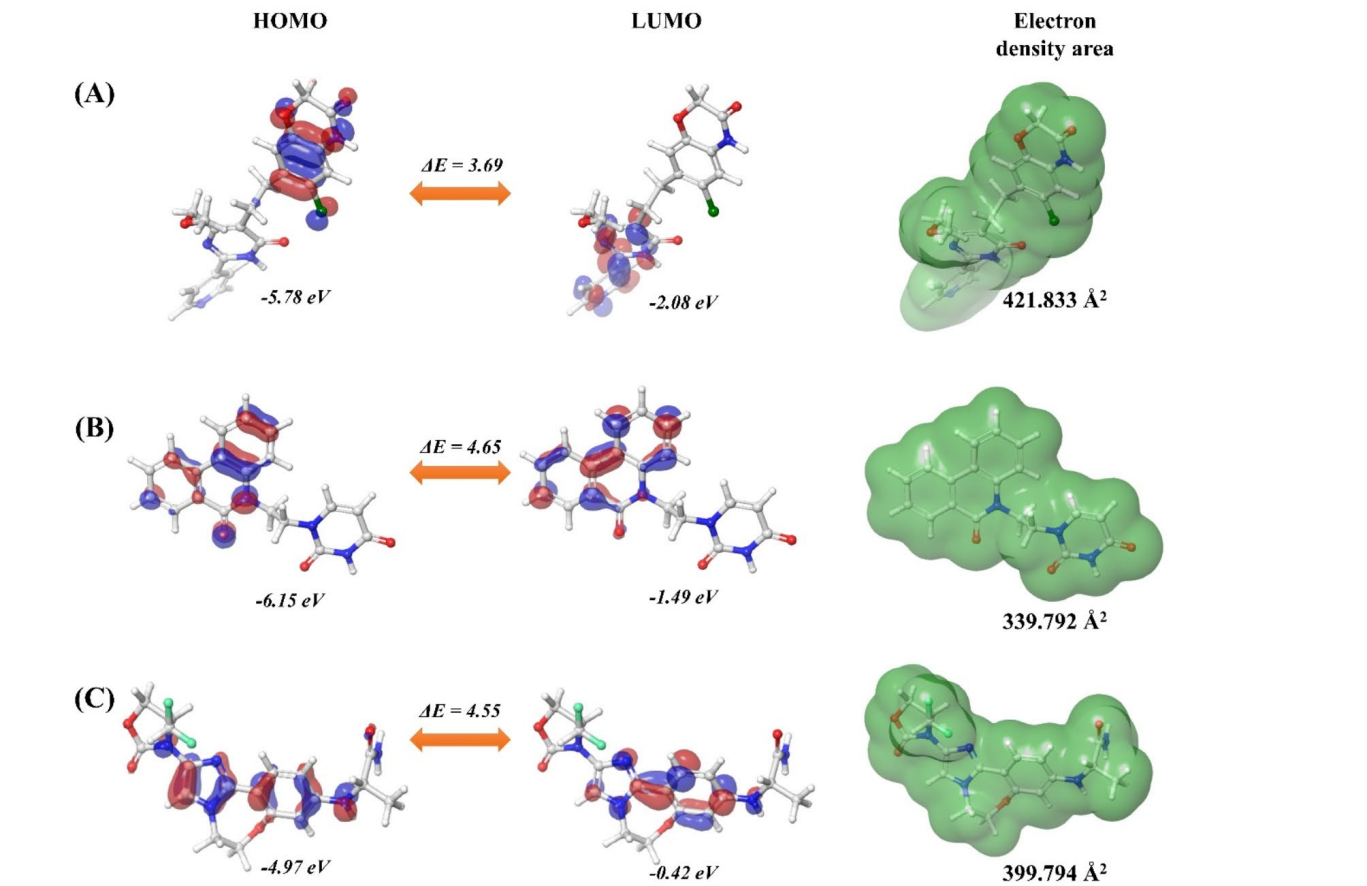
The contour mapping of the HOMO–LUMO states and electron density of the top two compounds – Djh1 and Djh2, along with Inavolisib.

Further, the HOMO–LUMO state of the top two compounds- Djh1, and Djh2, along with Inavolisib were calculated using the DFT function, and the contour mapping was reported in the Fig. 9.

It was observed that the electron density area of the top two compounds was in the similar range of the standard PI3Kα inhibitor Inavolisib. Along with that, the stability of the top two molecules Djh1 and Djh2 (i.e., ΔE) is also in the similar range as Inavolisib. The detailed quantum chemical descriptors of the top two compounds were compared with the Inavolisib in Table 4. The results were represented in the *eV* (electron volt), as it was converted from hartrees to *eV* (1 *hartree* = 27.2114 eV).

**Table 4:** Analysis of the quantum chemical descriptors of the top two compounds – Djh1 and Djh2, along with Inavolisib.

Compounds	HOMO	LUMO	ΔE	I	A	χ	Μ	η	Σ	ω
Djh1	−5.78406	−2.0863	3.697757	5.784055	2.086298	3.935177	−3.93518	1.848879	0.540868	4.18784
Djh2	−6.15631	−1.49962	4.656687	6.156307	1.49962	3.827964	−3.82796	2.328343	0.42949	3.146724
Inavolisib	−4.97288	−0.42259	4.55029	4.972883	0.422593	2.697738	−2.69774	2.275145	0.439532	1.599413

Therefore, the dynamic behavior of the ligand-receptor complex will provide insight into the stability and binding interaction-inhibition relationship.

### Simulation of ligand-receptor complex with dynamic behavior

The simulation of the ligand–protein complex using the Desmond module provides insight into the binding relationship with specific amino acids, along with the stability of the ligand at the catalytic binding site. The solvated model in the orthorhombic box can imitate the biological characteristics with extreme significance, as IFD and DFT calculations were unable to execute the simulation throughout 100 ns. Further, the RMSD, RMSF, and binding site analysis for inhibitory activity were represented in the study.

**Fig. 10:**
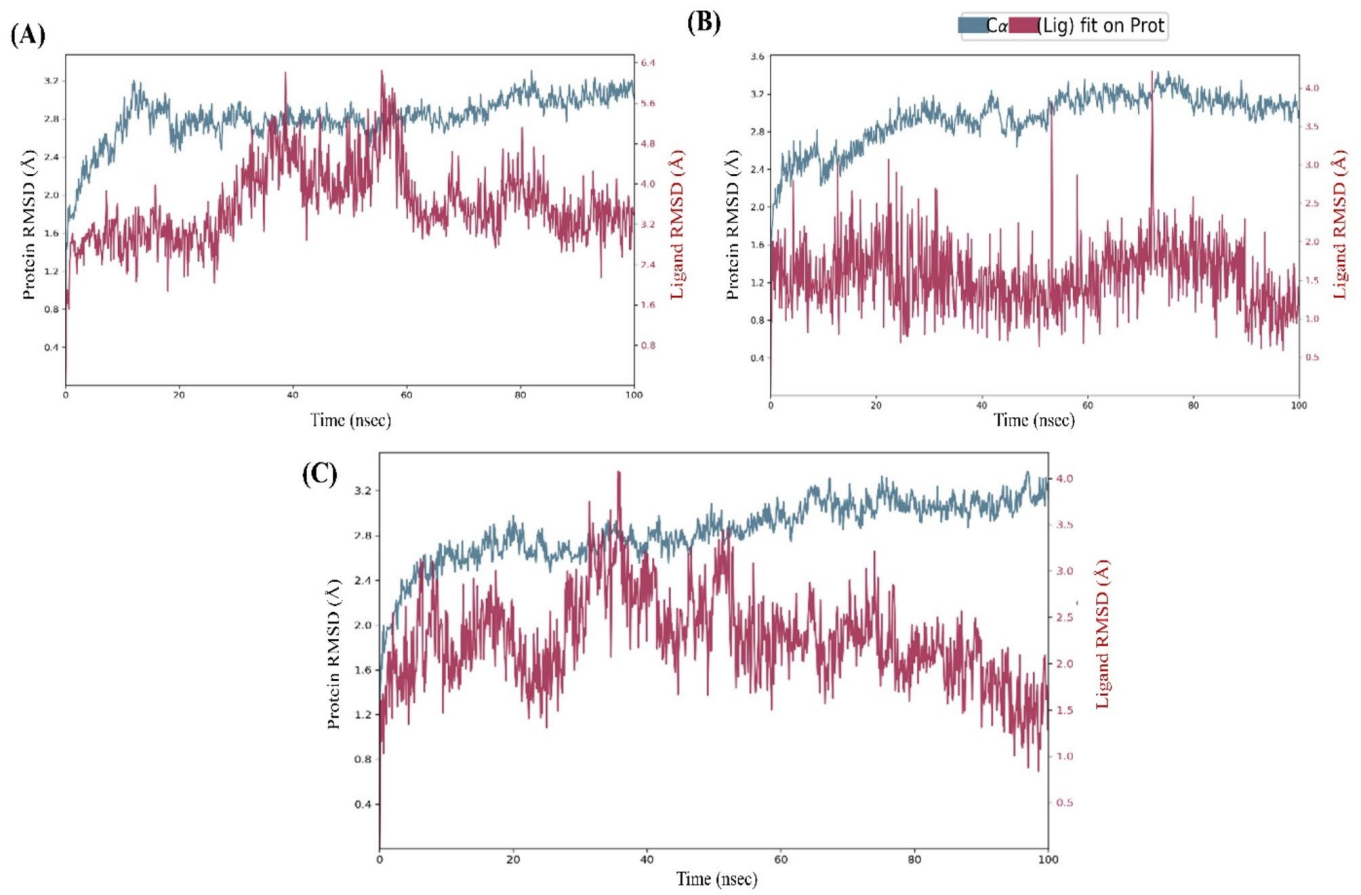
RMSD of the Complex (**A**), Complex (**B**), and Complex (**C**) – i.e., PI3Kα-Djh1, PI3Kα-Djh2, and PI3Kα-Inavolisib, respectively.

The root mean square deviation (RMSD) of Complex (A) was stable with an average receptor backbone of 2.80 Å, an average ligand RMSD of 3.59 Å and Complex (B) with an average receptor backbone of 2.95 Å, and an average ligand RMSD of 1.53 Å. Hence, the Complex (A) PI3Kα – Djh1 and Complex (B) PI3Kα-Djh2 were stable when compared with the Complex (C) PI3Kα-Inavolisib – with an average receptor backbone of 2.25 Å, and average ligand RMSD of 2.83 Å. Further, the fluctuations of the RMSD of Complex (A) and Complex (B) was less than those of Complex (C). Hence, the Complex (A) PI3Kα – Djh1 and Complex (B) PI3Kα-Djh2 represented excellent compatibility and stability throughout the 100 ns simulation, reported in Fig. 10.

**Fig. 11:**
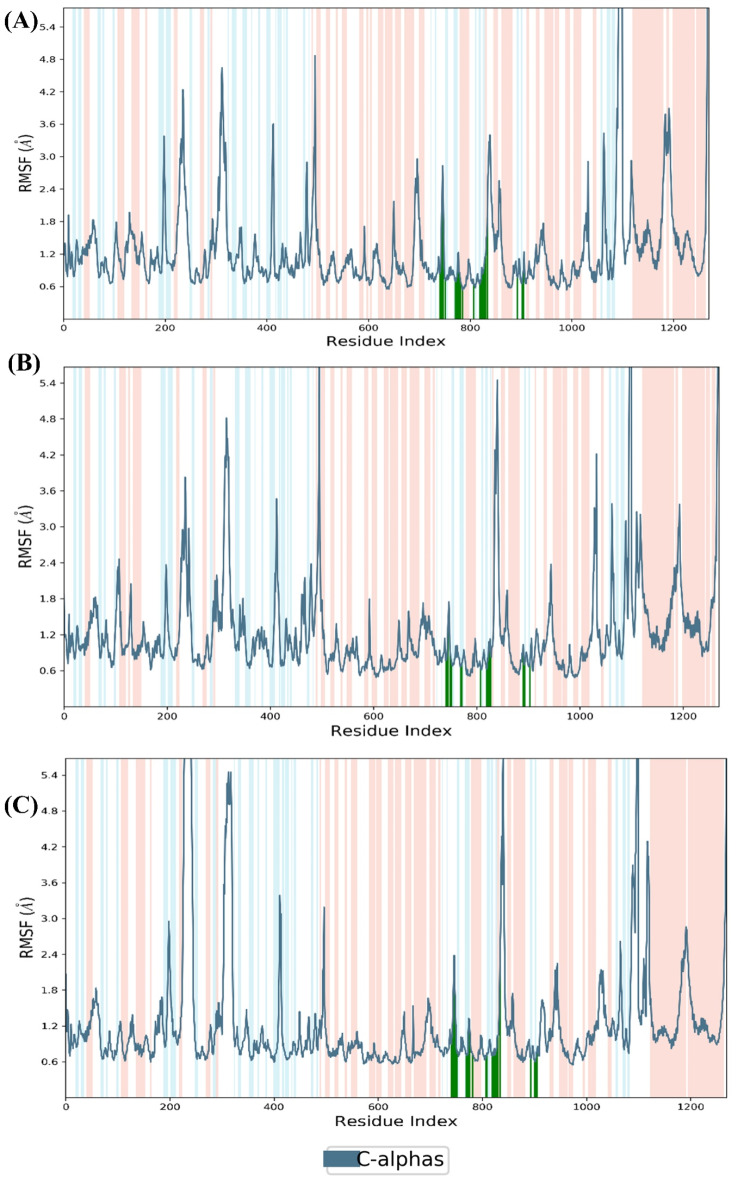
RMSF of the Complex (**A**), Complex (**B**), and Complex (**C**) – i.e., PI3Kα-Djh1, PI3Kα-Djh2, and PI3Kα-Inavolisib, respectively.

The average fluctuations of the protein [i.e., root mean square fluctuations (RMSF)] of PI3Kα-Complex (A) was 1.27 Å, and PI3Kα-Complex (B) was 1.22 Å, while the PI3Kα-Complex (C) was 1.12 Å. Hence, it was observed that the protein was extremely stable throughout the simulation with all three compounds – Djh1, Djh2, and Inavolisib. The flexibility of the protein in the α-helix and β-sheet was less in Complex (A) and Complex (B) rather than in Complex (C). The fluctuations of Complex (A) and (B) in the vacant region were less than the Complex (C). The strong contacts of the ligands and protein were reported in Fig. 11, and it was observed that Complex (A) and (B) of Djh1 and Djh2 showed similar contacts in Complex (C) of Inavolisib for inhibitory efficacy at the catalytic binding site.

**Fig. 12:**
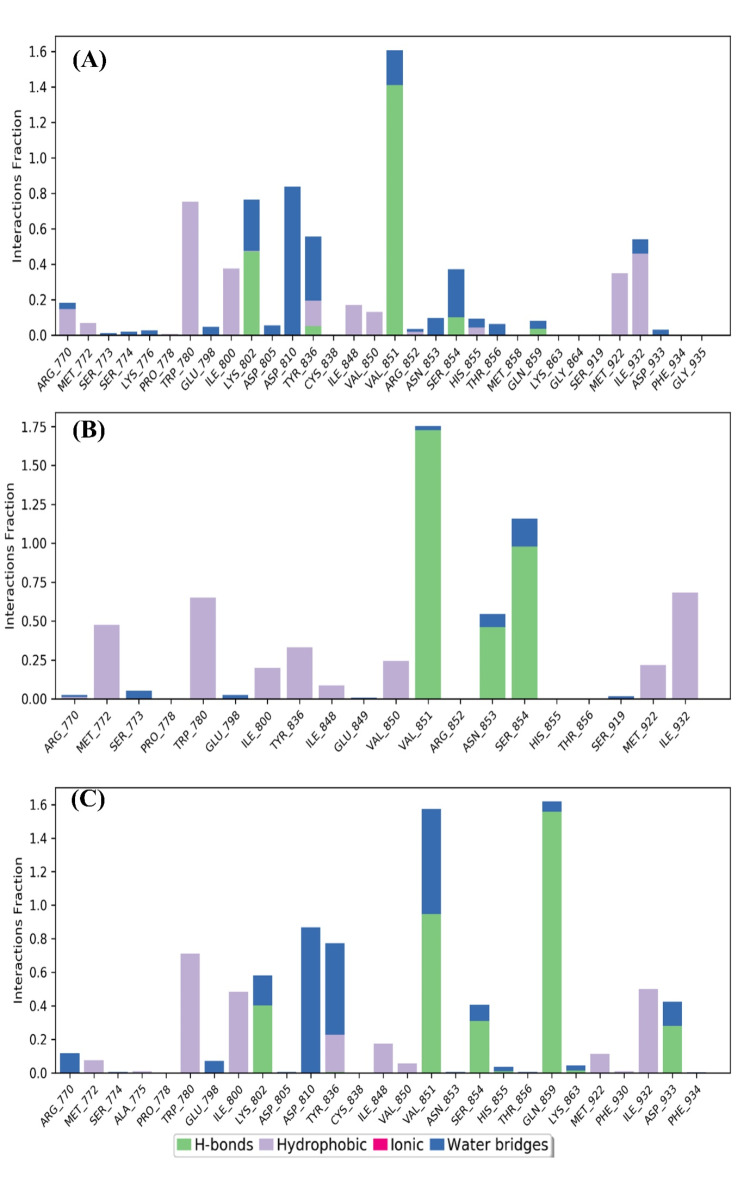
Histogram of the Complex (**A**), Complex (**B**), and Complex (**C**) – i.e., PI3Kα-Djh1, PI3Kα-Djh2, and PI3Kα-Inavolisib contacts, respectively.

**Fig. 13:**
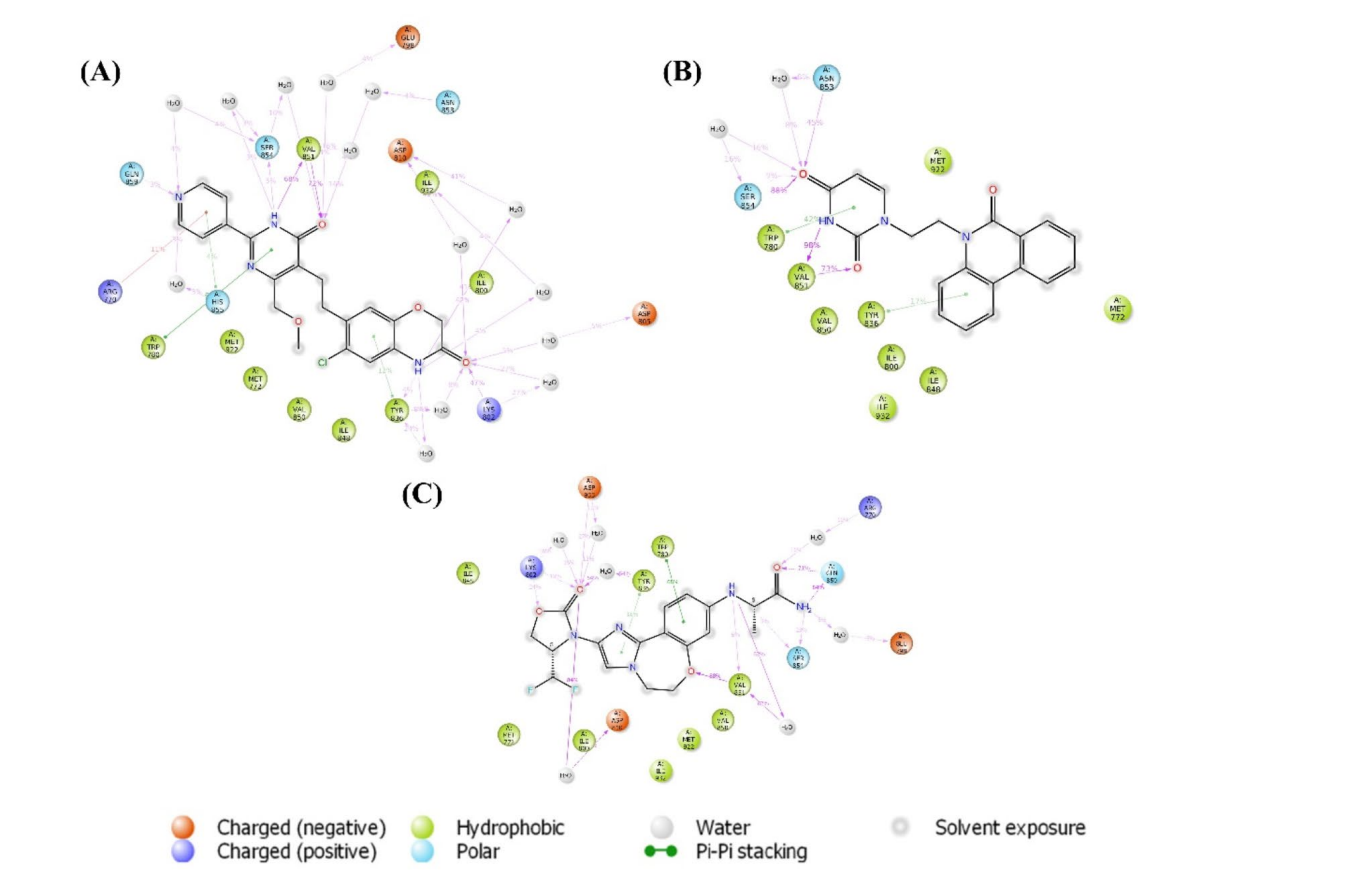
Contacts of Ligand-PI3Kα complex – (**A**) Djh1, (**B**) Djh2, and (**C**) Inavolisib.

The binding interaction of the molecule and Receptor plays a significant role in the inhibition of the specific protein, similar to PI3Kα, by contact with important amino acid residues like Valine 851 and Serine 854. Hence, throughout the 100 ns simulation, the binding interaction of Djh1, Djh2, and Inavolisib represented a crucial role in Complex (A), Complex (B), and Complex (C), respectively. The interaction of the hit compounds – Djh1 and Djh2 showed Hydrogen bonds with water bridges (the best interaction of Desmond simulation using OPLS4 force field) with Valine 851 amino acid residue similar to PI3Kα inhibitor Inavolisib. The compounds also showed H-bond interaction with Serine 854, and it can be concluded that the top two hit compounds will act as selective PI3Kα inhibitors, as they represented ligand-receptor contacts similar to Inavolisib throughout the simulation. The histogram of the ligand–protein contacts, along with the ligand–protein binding 2D interaction diagram, was reported in Figs. 12 and 13, respectively. The compounds – Djh1 and Djh2 can act as selective PI3Kα inhibitors for the treatment of TNBC and also act as a potential lead for the design of new molecules through bioisosteric replacement. Further, the multivariate statistical analysis was provided in the Supplementary Figure S20-S25.

### Bioisosteric replacement

**Fig. 14:**
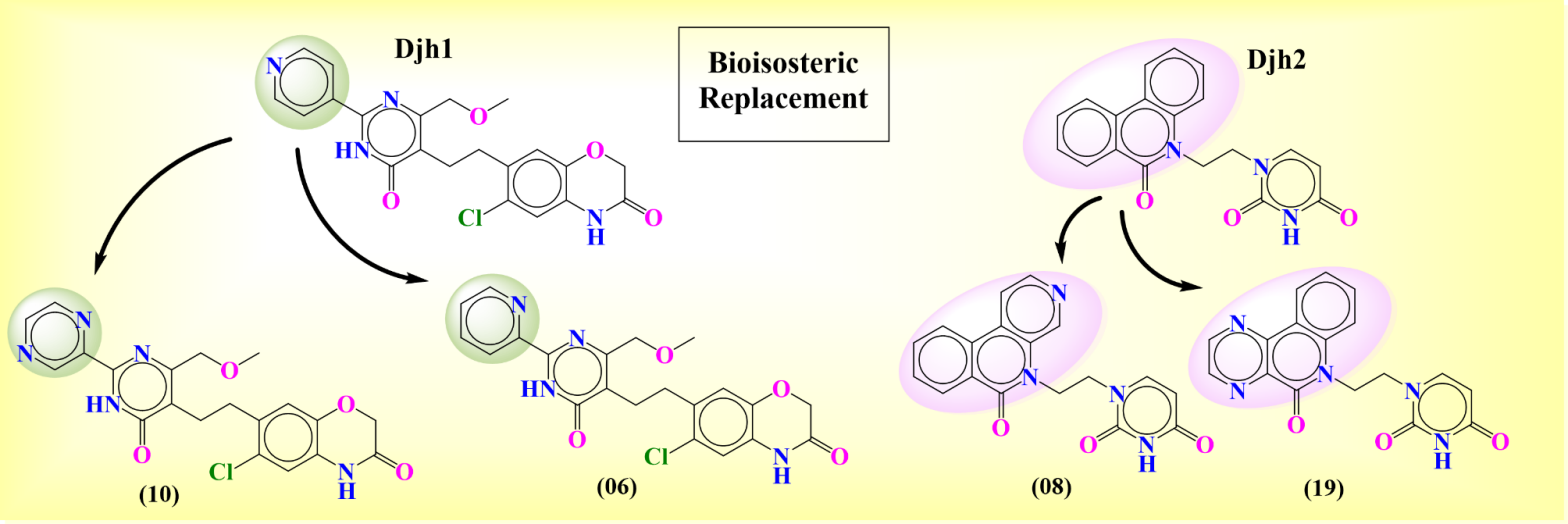
The best two bioisosteres of the top two compounds – Djh1, and Djh2 – Compound 10, Compound 6, and Compound 8, Compound 19, respectively.

The two compounds – Djh1 and Djh2, represented excellent stability and interaction in PI3Kα inhibition for anticancer activity against TNBC in the molecular dynamics simulation. Here, we utilized the bioisosteric replacement tool of Maestro to develop novel moieties for better binding interactions with similar selectivity towards PI3Kα and ADMET properties. The bioisosteric replacement of Djh1 and Djh2 was reported in Fig. 14. The compound Djh1 generated ten bioisosteres, and Djh2 generated 21 bioisosteres. The molecular docking analysis showed a better docking score and similar binding interaction for compounds 10 and 6 for Djh1 and compound 8 and compound 16 for Djh2, as reported in Table 5. The pyridine moiety with nitrogen at the para position of Djh1 was replaced with pyrazine with nitrogen at 2 and 5 positions,—termed compound 10, and pyridine with nitrogen at position 2 was termed compound 6. Similarly, the change of nitrogen position and addition of more nitrogen in the nitro-phenanthrene moiety leads to the development of compound 8 and compound 9 from Djh2.

**Table 5:** The top two compounds of bioisosteres – Djh1 [Compound 10, Compound 6] and Djh2 [Compound 08, Compound 19] 2D interaction diagram with target selectivity analysis.

Compound ID		Interaction diagram (2D)	XP Docking score (kcal/mol)	Important interactions	Target selectivity analysis
Djh1	10		−10.947	**H-bond:** V851, N853, **π-π stacking:** H855, W780	
	06		−10.727	**H-bond:** V851, Q859, Y836, **π-π stacking:** H855, W780, Y836	
Djh2	08		−10.515	**H-bond:** V851, Q859, **π-π stacking:** Y836	
	19		−10.305	**H-bond:** V851, K802, **π-π stacking:** W780	
**[Note:** A (Alanine), R (Arginine), N (Asparagine), D (Aspartic acid), C (Cysteine), E (Glutamic acid), Q (Glutamine), G (Glycine), H (Histidine), I (Isoleucine), L (Leucine), K (Lysine), M (Methionine), F (Phenylalanine), P (Proline), S (Serine), T (Threonine), W (Tryptophan), Y (Tyrosine), V (Valine)**]**					

The ligand-receptor docking analysis represented that compound 10 and compound 6 represented better docking score of −10.947 kcal/mol and −10.727 kcal/mol, respectively, than Djh1, although similar binding interaction in its parent moiety Djh1; compound 8 and compound 19 represented better docking score of −10.515 kcal/mol and −10.305 kcal/mol respectively than Djh2, although similar binding properties alike its parent moiety Djh2. There was also no significant decrease in the kinase selectivity of the bioisosteres. Hence, the bioisosteres will also provide novel therapeutics for selective PI3Kα inhibition in the treatment of TNBC. Further ADMET analysis will provide more insight into whether the compounds – Djh1, Djh2, and their top two bioisosteres – were druggable moieties and their pharmacokinetic properties. The 3D interaction diagram was reported in the Supplementary Figure S15- S18.

### Druglikeness and ADMET analysis

Following the fragment-based design protocol for novel molecule designing and getting hit molecules by molecular docking, MMGBSA, IFD analysis, DFT studies, and simulation of ligand–protein complex,—drug-likeness and ADMET analysis are necessary for in vitro and in vivo application. Therefore, to increase human compatibility and better pharmacokinetic profiling without losing kinase selectivity, a bioisosteric replacement was employed for hit-to-lead optimization. The drug-likeness properties of Djh1 and Djh2, along with their bioisosteres – compounds 10 and 06, compounds 08 and 19, respectively, were analyzed using the QikProp module of Maestro while comparing with Inavolisib, reported in Table 6. Further, the ADMET profiling was analyzed in an extensive way, using the PkCSM open server, reported in Supplementary Table S3, and toxicity analysis was reported in Table 7, which provided an excellent analysis of the molecules, which might be a potential lead in the treatment of TNBC by selectively targeting PI3Kα.

**Table 6:** Druglikeness by QikProp module of the top two compounds – Djh1 and Djh2, along with bioisosteres—Compound 10, Compound 06, and Compound 08, Compound 19, respectively.

Compounds	Molecular weight	Hydrogen bond donor	Hydrogen bond Acceptor	QPlogP (o/w)	Percent human oral absorption	Rule of five violations
Djh1	426.858	2.000	9.950	1.989	80.898	0
10	427.846	2.000	10.950	1.367	81.847	0
06	426.858	2.000	9.450	2.466	88.436	0
Dhj2	333.346	1.000	6.500	2.103	81.305	0
08	334.334	1.000	8.000	1.158	89.649	0
19	335.321	1.000	8.500	0.923	88.861	0
Inavolisib	407.376	3.000	9.250	1.137	71.655	0

**Table 7:** Detailed toxicity analysis by pkCSM.

Compounds	Toxicity										
	Renal OCT2 substrate (Yes/No)	AMES toxicity (Yes/No)	Max tolerated dose (human) (log mg/kg/day)	hERG I inhibitor (Yes/No)	hERG II inhibitor (Yes/No)	Oral rat acute toxicity (LD50) (mol/kg)	Oral Rat Chronic Toxicity (LOAEL) (log mg/kg_bw/day)	Hepatotoxicity (Yes/No)	Skin Sensitisation (Yes/No)	*T. Pyriformis* toxicity (log μg/L)	Minnow toxicity (log mM)
Djh1	No	No	0.111	No	Yes	2.594	1.38	Yes	No	0.294	−0.016
10	No	No	0.287	No	Yes	2.303	1.469	Yes	No	0.289	0.01
06	No	No	0.098	No	Yes	2.591	1.356	Yes	No	0.299	0.172
Dhj2	No	Yes	0.147	No	Yes	2.78	1.899	Yes	No	0.304	2.2
08	No	Yes	−0.3	No	Yes	2.895	1.286	Yes	No	0.292	3.35
19	No	Yes	0.433	No	Yes	2.798	1.296	Yes	No	0.286	2.746
Inavolisib	No	Yes	0.044	No	Yes	2.507	1.269	Yes	No	0.285	2.434

**Fig. 15:**
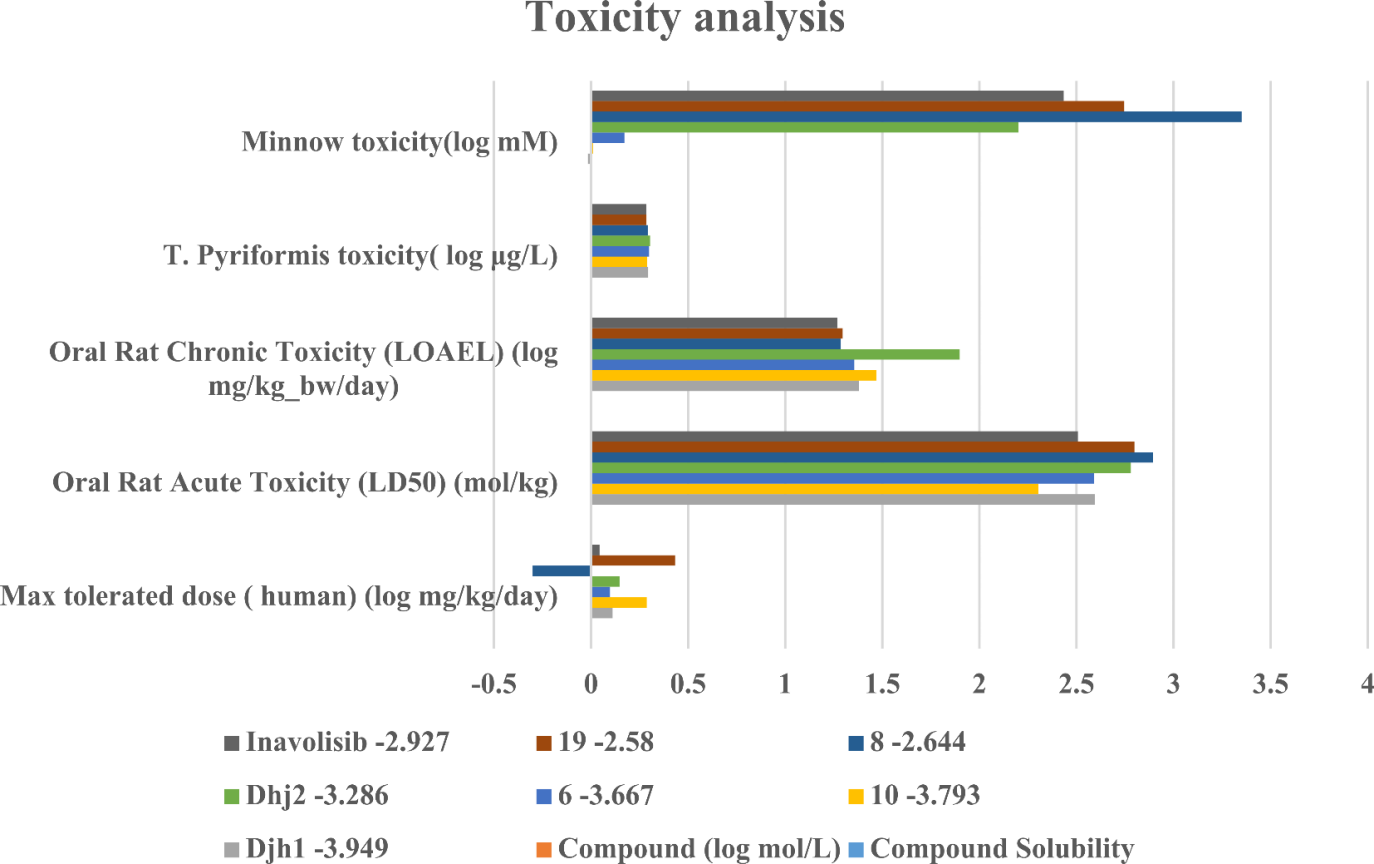
Graphical representation of the Toxicity analysis of compounds- Djh1 and its bioisosteres – Compound 10 and Compound 06, Djh2 and its bioisosteres – Compound 08 and Compound 19.

While comparing the Absorption, Distribution, Metabolism, Excretion, and Toxicity (ADMET) descriptors with the selective PI3Kα inhibitor Inavolisib, the hit compounds – Djh1 and Djh2, along with their bioisosteres – compound 10 and 6, and compound 8 and 19 represented drug-likeness by not violating the Lipinski rule of five. The ADMET descriptors also were in a similar range to the selective PI3Kα inhibitor Inavolisib. The toxicity profiling was analyzed, and it was observed that the molecules might be hepatotoxic in nature, so oral administration must be avoided. Several other toxicity parameters,—AMES toxicity, hERG I and hERG II inhibition, Oral acute rat toxicity, etc., were measured and analyzed. A graphical analysis was also provided in Fig. 15. Hence, the compounds can act as potential leads for anticancer therapeutics in vitro and in vivo analysis. Further structure–activity relationship of the top compound – Djh1, was reported on the basis of the in silico analysis.

### Structure–activity relationship analysis

**Fig. 16:**
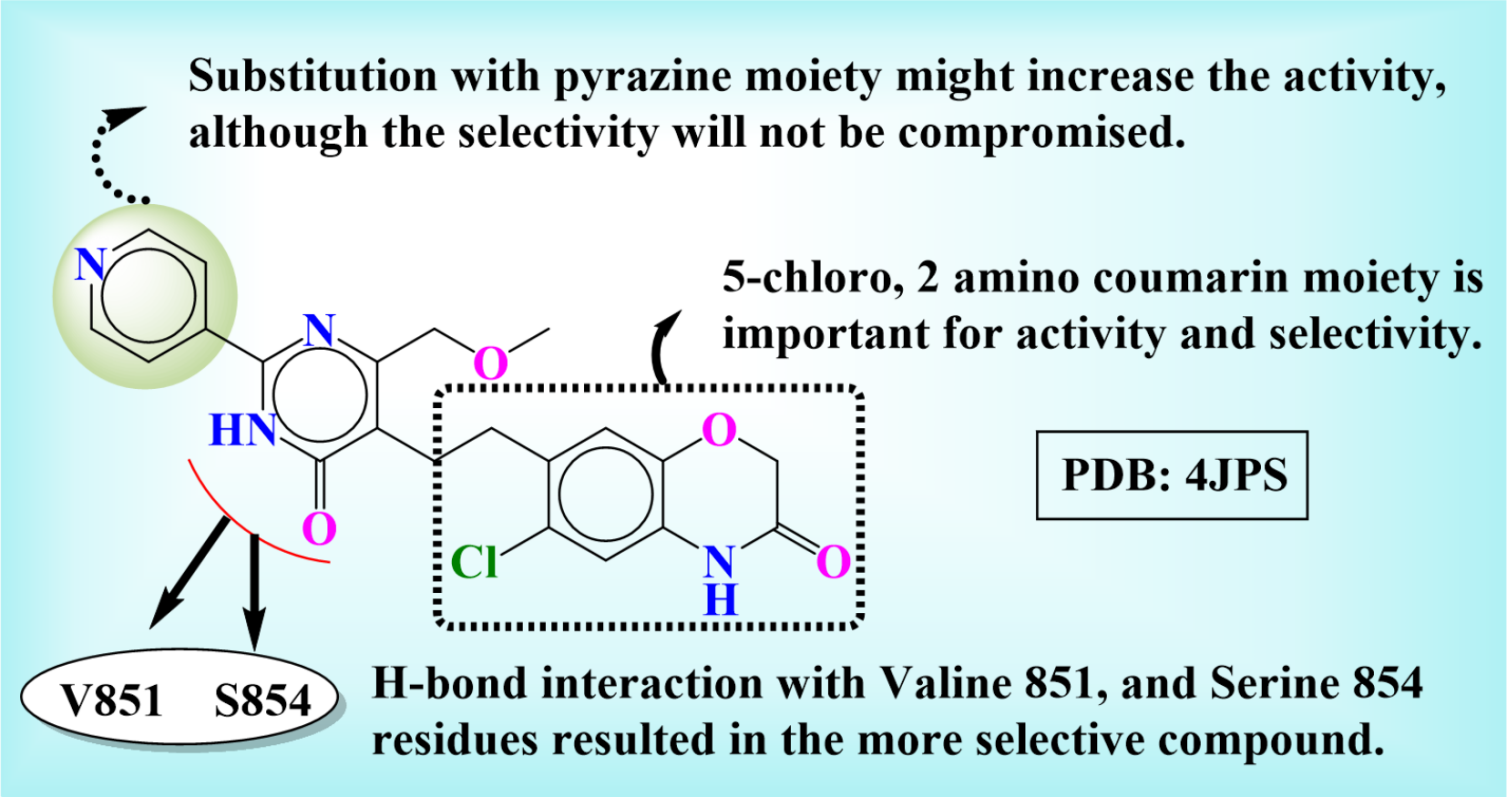
In silico SAR of the compound – Djh1.

The structure–activity relationship signifies the overall study findings from the medicinal chemistry perspective, as it is a bridge between computational, organic, and biological chemistry. The present study analyses the hit compound – Djh1 and its bioisosteres to be potential candidates for in vitro and in vivo activity targeting selectively PI3Kα for TNBC treatment. The 5-chloro, 2-amino coumarin moiety is important for activity and selectivity towards PI3Kα as out of the top ten molecules after molecular docking and MMGBSA analysis, and eight molecules contain the same moiety. Hence, the amino coumarin moiety might be a potential lead moiety to develop novel compounds for PI3Kα selective inhibition. Substituting pyrazine moiety in place of pyridine might increase the activity, although selectivity will not be compromised. In conclusion, the core amino acid residues responsible for PI3Kα selective inhibition were Valine 851 and Serine 854, and the H-bond interaction with the amide of pyrimidine ring is the most important for selective inhibition of PI3Kα as potential lead for cancer treatment, especially in TNBC, reported in Fig. 16.

## Conclusion

Triple-negative breast cancer is one of the most pernicious cancers globally, and the available drugs are growing resistant day-by-day. Multi-drug resistance due to chemotherapy and radiation therapy, poor patient compliance, and the need for novel molecules for the treatment of uncontrolled cell proliferation, as well as mutation in TNBC, urged the contemporary study. The quest for novel molecules with the integration of fragment-based approach, scaffold hopping, and virtual screening with structure-based molecular docking and MMGBSA analysis has already been proven as a powerful and synergistic tool for novel therapeutics in the treatment of TNBC targeting selectively PI3Kα. Utilizing the 11,269 fragments as building blocks from the ChemDiv database for novel molecule design targeting PI3Kα,—100 novel molecules were generated following the Combine Fragments protocol. Further, the ligand-receptor docking and target selectivity analysis provided the outcome that fragments with 5-chloro, 2-amino coumarin moiety with ethyl linker should be potential candidates in selective inhibition of PI3Kα. Hence, the integration of virtual screening provides insights into the rational drug design and amino acid residues that are significant in the inhibition at the catalytic binding site. Despite the challenges of the false positive outcomes, MMGBSA free binding energy analysis was executed with top 10 compounds – Djh1 – Djh10. Following the outcomes of binding interactions, docking score, and target selectivity analysis, the top two molecules – Djh1 and Djh2 were selected for further analysis of IFD, DFT, and molecular dynamics simulation. The analysis of IFD and dynamic simulation of the PI3Kα-Djh1 and PI3Kα- Djh2 complex provided excellent stability and binding affinity for the selective inhibition of the PIK3CA gene by interacting with Valine 851 and Serine 854 at the catalytic binding site. The stability of the compounds,—Djh1 and Djh2 were also analyzed using the HOMO–LUMO analysis and other quantum chemical descriptors, which provided a similar range of outcomes to Inavolisib. Therefore, to design more novel moieties, we have incorporated the bioisosteric replacement tool of Maestro to generate more novel moieties of the top two compounds – Djh1 and Djh2. Further docking and target selectivity analysis provided outcomes of compound 10 and compound 9 for Djh1, compound 8, and compound 19 for Djh2 with better dock score and similar ADMET properties like the parent moiety. While comparing with selective PI3Kα inhibitor Inavolisib, it was observed that Djh1 and Djh2 reported similar or better results in the docking analysis, induced fit analysis, density function theory analysis, and dynamic simulation study. In the case of bioisosteres, it also did not significantly decrease the selectivity, although it increases the binding affinity for the treatment of TNBC.

Due to the small number of drugs targeting PI3Kα that are covered in this investigation, there is a chance that some intriguing candidates with favorable pharmacological properties will be missed. The constrained chemical library might make it more difficult to explore structure–activity correlations (SAR), which would impede the optimization of lead compounds into more effective and targeted therapeutic prospects. Moreover, there are a number of limitations to computational virtual screening that may make the false negative issue worse. These limitations include the quantity and complexity of the chemical libraries that may be screened, which may be limited by restrictions in computational resources like processing power and memory. Furthermore, the quality of the structural and pharmacological data that is now accessible and the robustness of the computational techniques used determine the accuracy and dependability of screening algorithms. The possibility of false negative results might be increased by biases or errors introduced by inadequate data sources or analytical approaches. A multimodal strategy is needed to address the issues of false negatives in computational virtual screening and the constraints related to creating a restricted number of chemicals.

Further, the top compounds’ in vitro and in vivo activity might provide potential leads for anticancer activity. The effective strategy of combined fragment-based drug design, structure-based screening, and bioisosteric replacement are complementary and offer a comprehensive toolkit to understand the complete landscape of molecular interactions. We expect that these protocols will speed up the process of anticancer drug development for a broad range of malignancies to overcome the problem of multi-drug resistance and poor patient compliance.

## Electronic Supplementary Material

Below is the link to the electronic supplementary material.

Supplementary Material 1

## Data Availability

The data that support the findings of this study are available from the corresponding author upon reasonable request.
