# Supplementary material for: Exploring target selectivity in designing and identifying PI3Kα inhibitors for triple negative breast cancer with fragment-based and bioisosteric replacement approach
Source: Sci Rep. 2025 Jan 13;15:1890. doi: 10.1038/s41598-024-83030-1 (PMC11729857; doi:10.1038/s41598-024-83030-1)
Supplement: Supplementary file 1 — Supplementary Material 1 [file 41598_2024_83030_MOESM1_ESM.docx]

**Exploring target selectivity in designing and identifying PI3Kα inhibitors for triple negative breast cancer with fragment-based and bioisosteric replacement approach**

Debojyoti Halder^1, *^, Shreya Mukherjee^1^, Jeyaprakash R S^1, **^

*^1^ Department of Pharmaceutical Chemistry, Manipal College of Pharmaceutical Sciences, Manipal Academy of Higher Education, Manipal-576104, Karnataka, India.*

***Corresponding author**

*Debojyoti Halder,*

*Department of Pharmaceutical Chemistry*

*Manipal College of Pharmaceutical Sciences,*

*Manipal Academy of Higher Education*

*Manipal, Karnataka-576104, India.*

*Email: debojyotihaldar955@gmail.com*

****Co-corresponding author**

*Jeyaprakash R S,*

*Department of Pharmaceutical Chemistry*

*Manipal College of Pharmaceutical Sciences*

*Manipal Academy of Higher Education*

*Manipal, Karnataka-576104, India.*

*Email: jeya.prakasham@manipal.edu*

**Supplementary Figures**


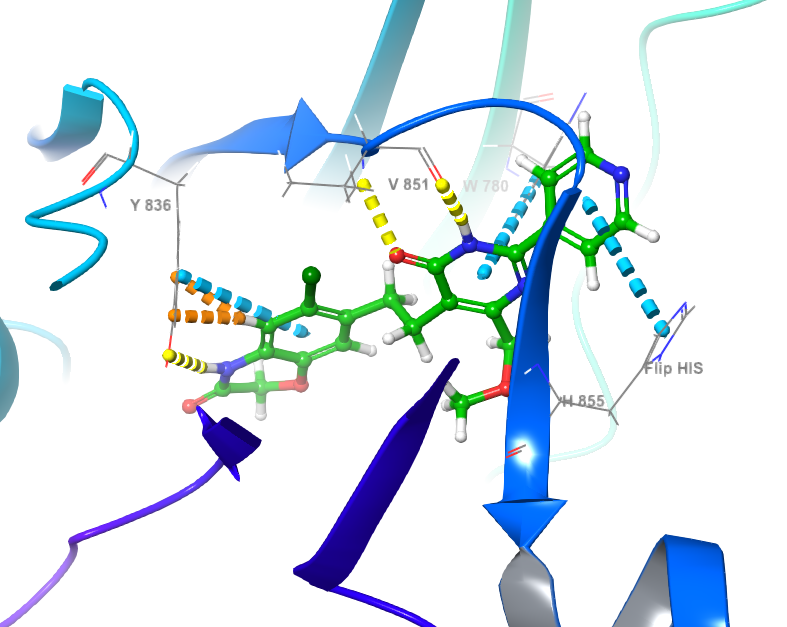


**Supplementary Figure S1.** 3D interaction diagram of Djh1 and PI3Kα


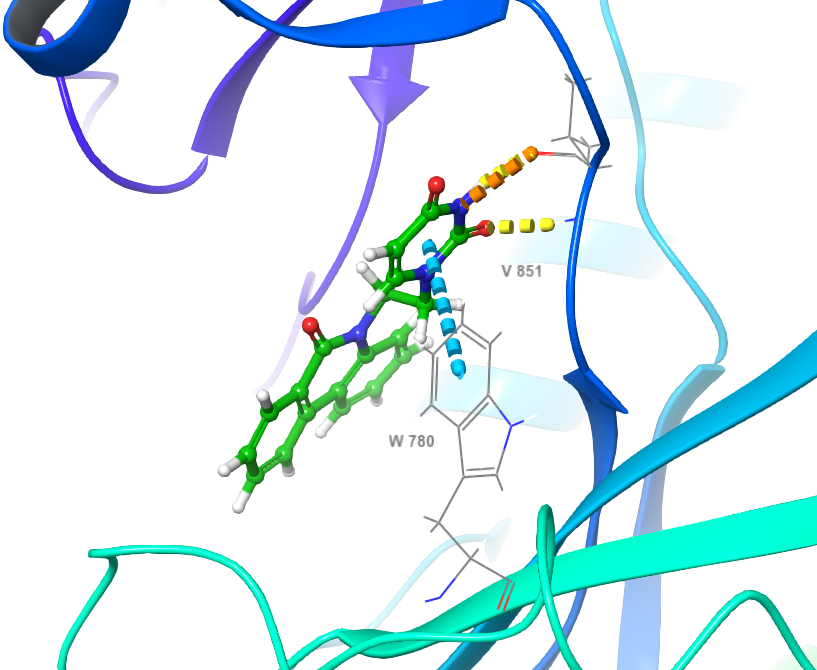


**Supplementary Figure S2.** 3D interaction diagram of Djh2 and PI3Kα


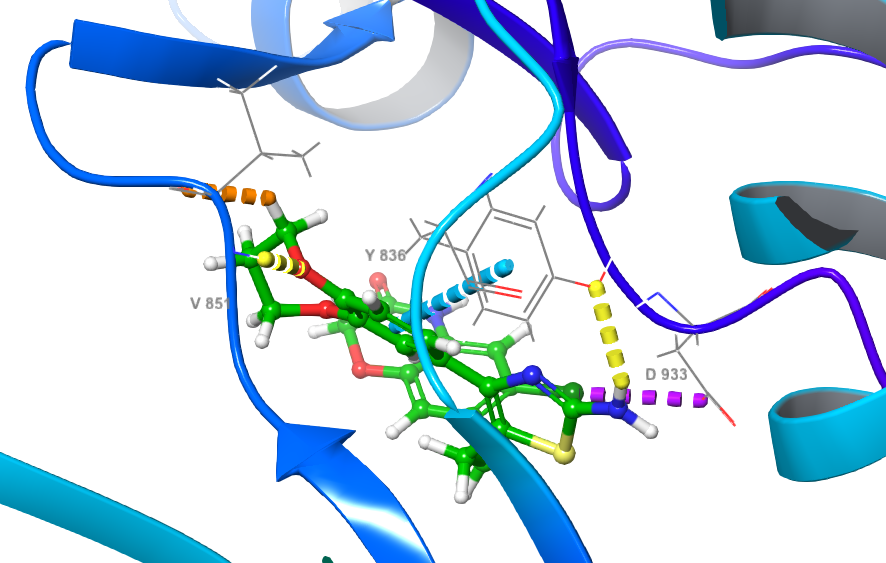


**Supplementary Figure S3.** 3D interaction diagram of Djh3 and PI3Kα.


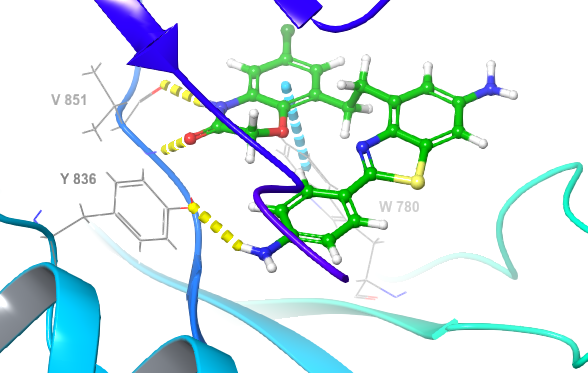


**Supplementary Figure S4.** 3D interaction diagram of Djh4 and PI3Kα.


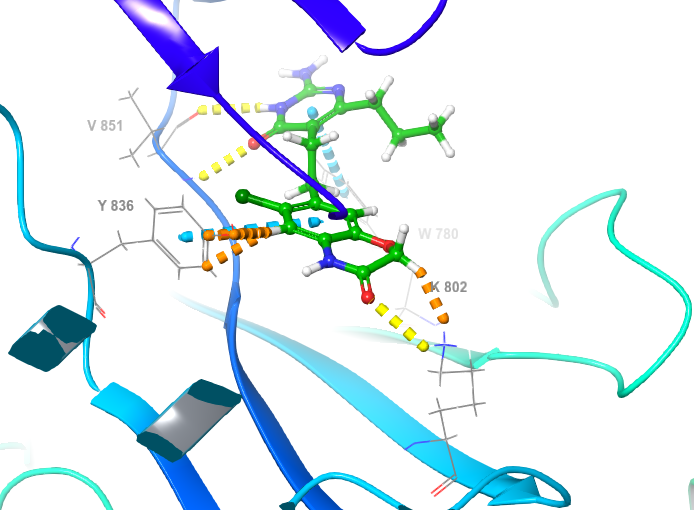


**Supplementary Figure S5.** 3D interaction diagram of Djh5 and PI3Kα.


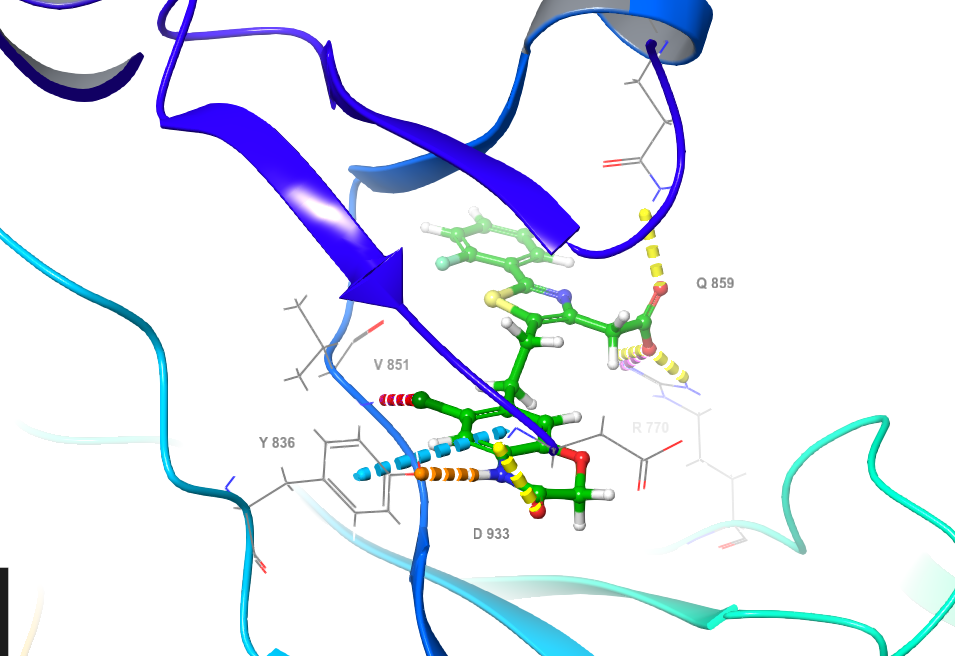


**Supplementary Figure S6.** 3D interaction diagram of Djh6 and PI3Kα.


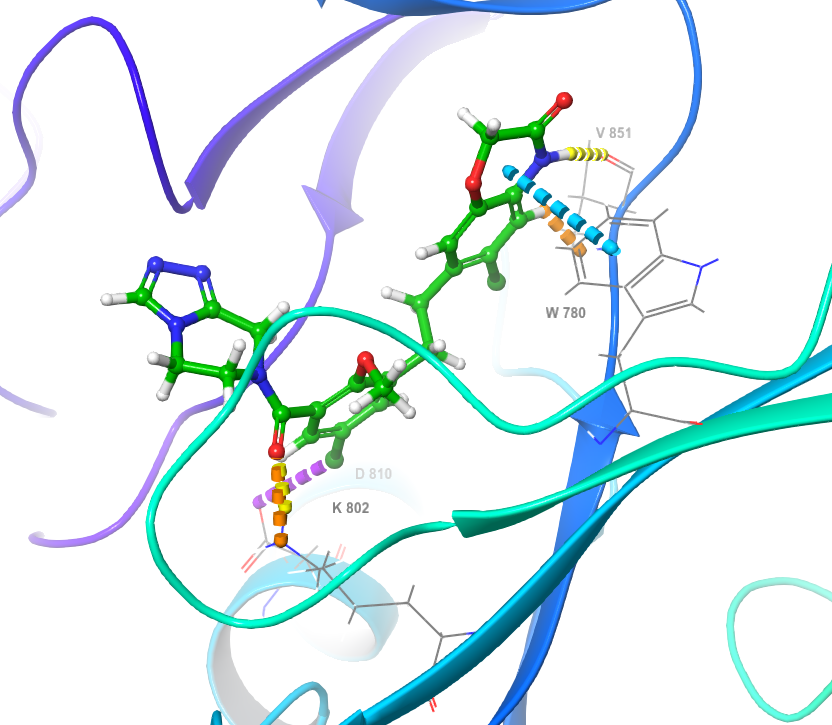


**Supplementary Figure S7.** 3D interaction diagram of Djh7 and PI3Kα.


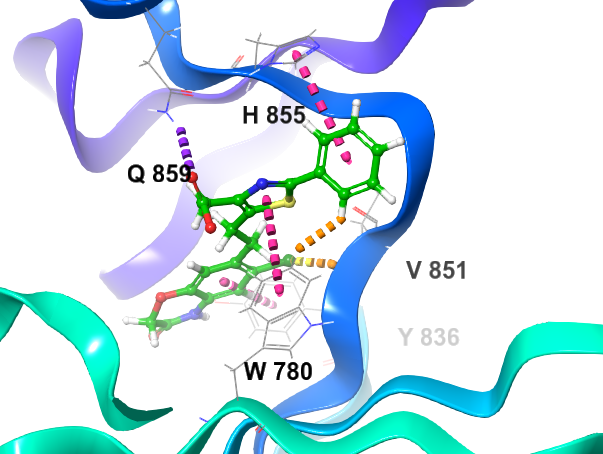


**Supplementary Figure S8.** 3D interaction diagram of Djh8 and PI3Kα.


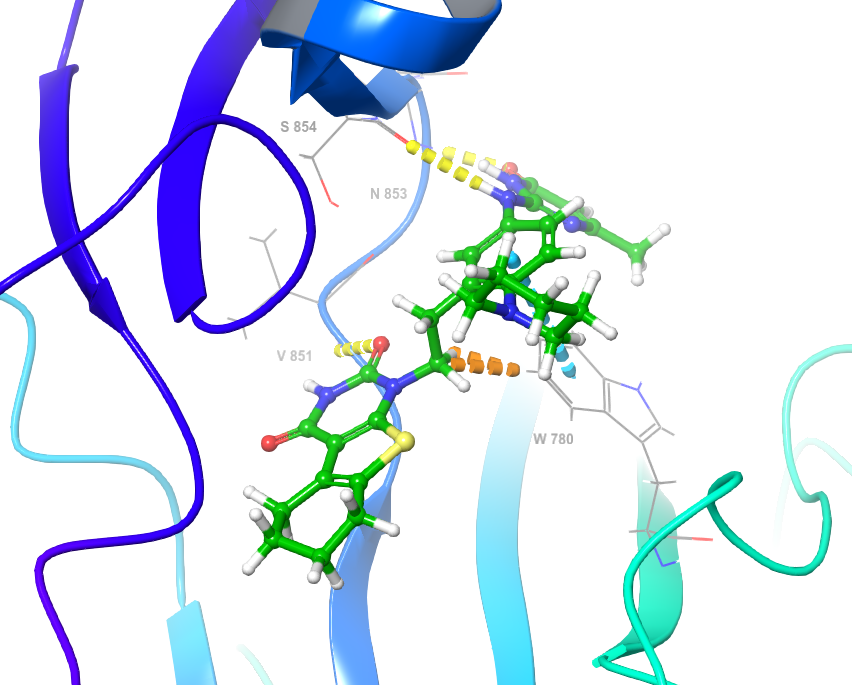


**Supplementary Figure S9.** 3D interaction diagram of Djh9 and PI3Kα.


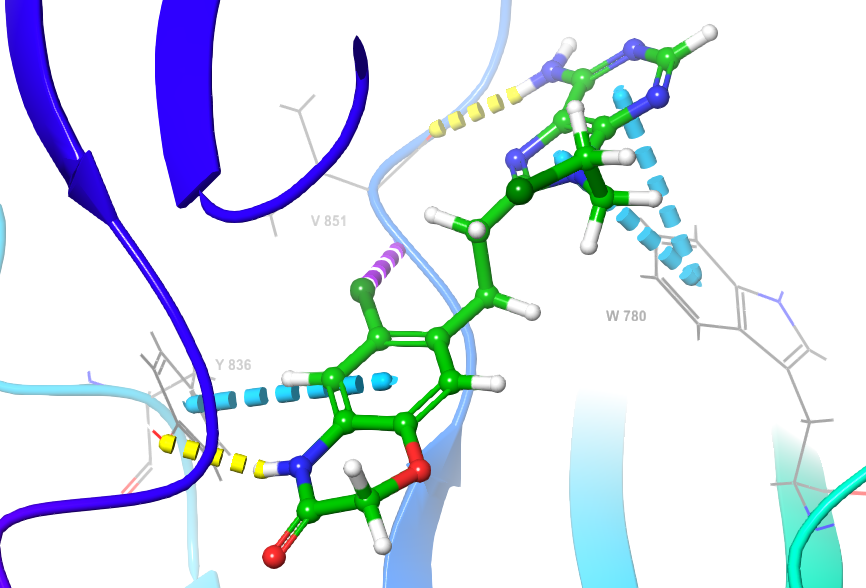


**Supplementary Figure S10.** 3D interaction diagram of Djh10 and PI3Kα.


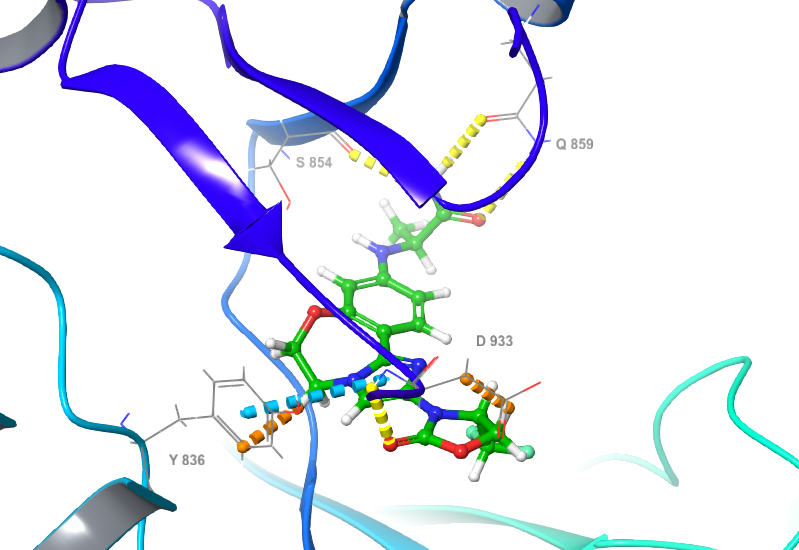


**Supplementary Figure S11.** 3D interaction diagram of Inavolisib and PI3Kα.


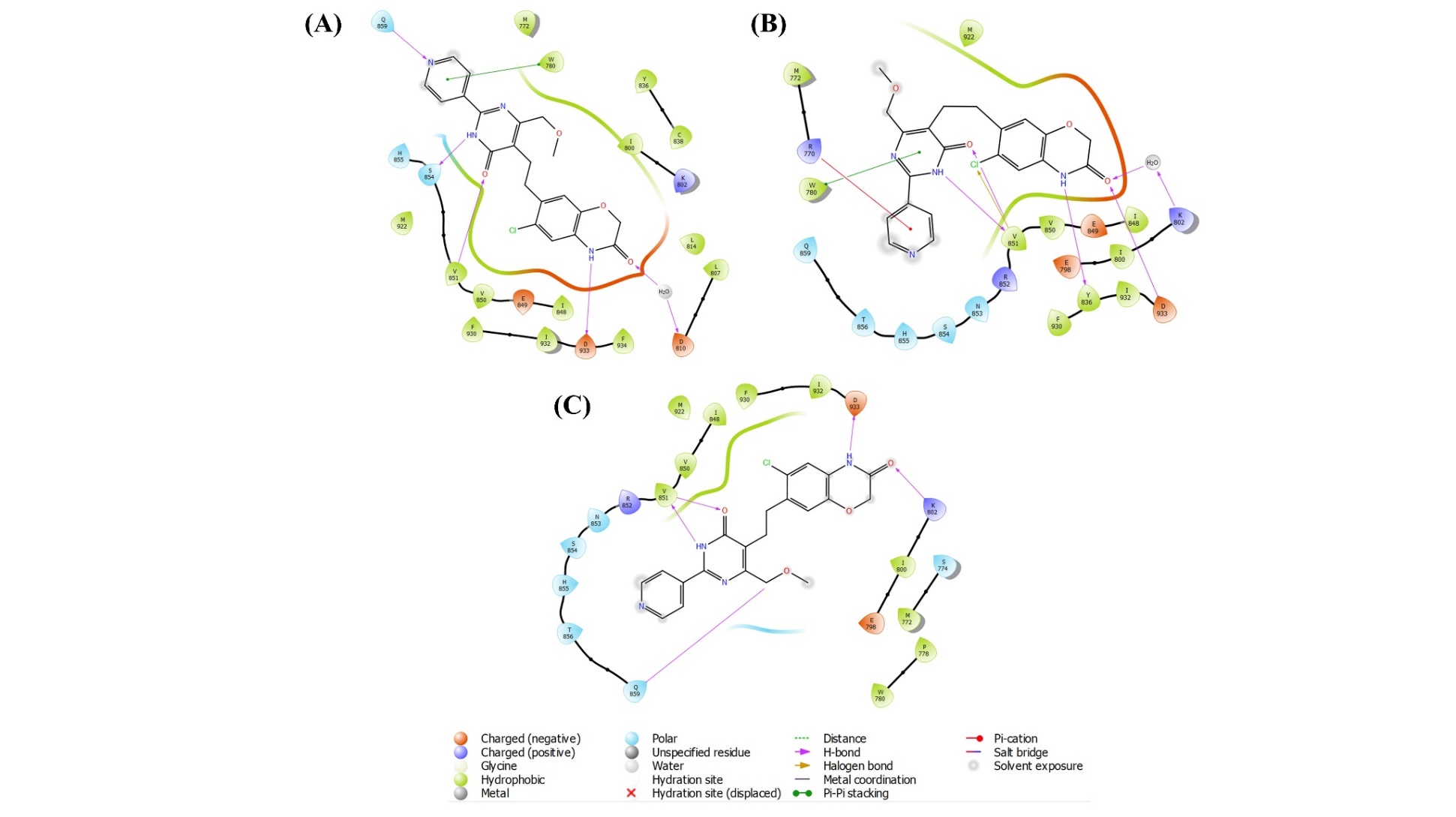


**Supplementary Figure S12.** 2D interaction diagram (IFD of top 3 poses) of Djh1 and PI3Kα.


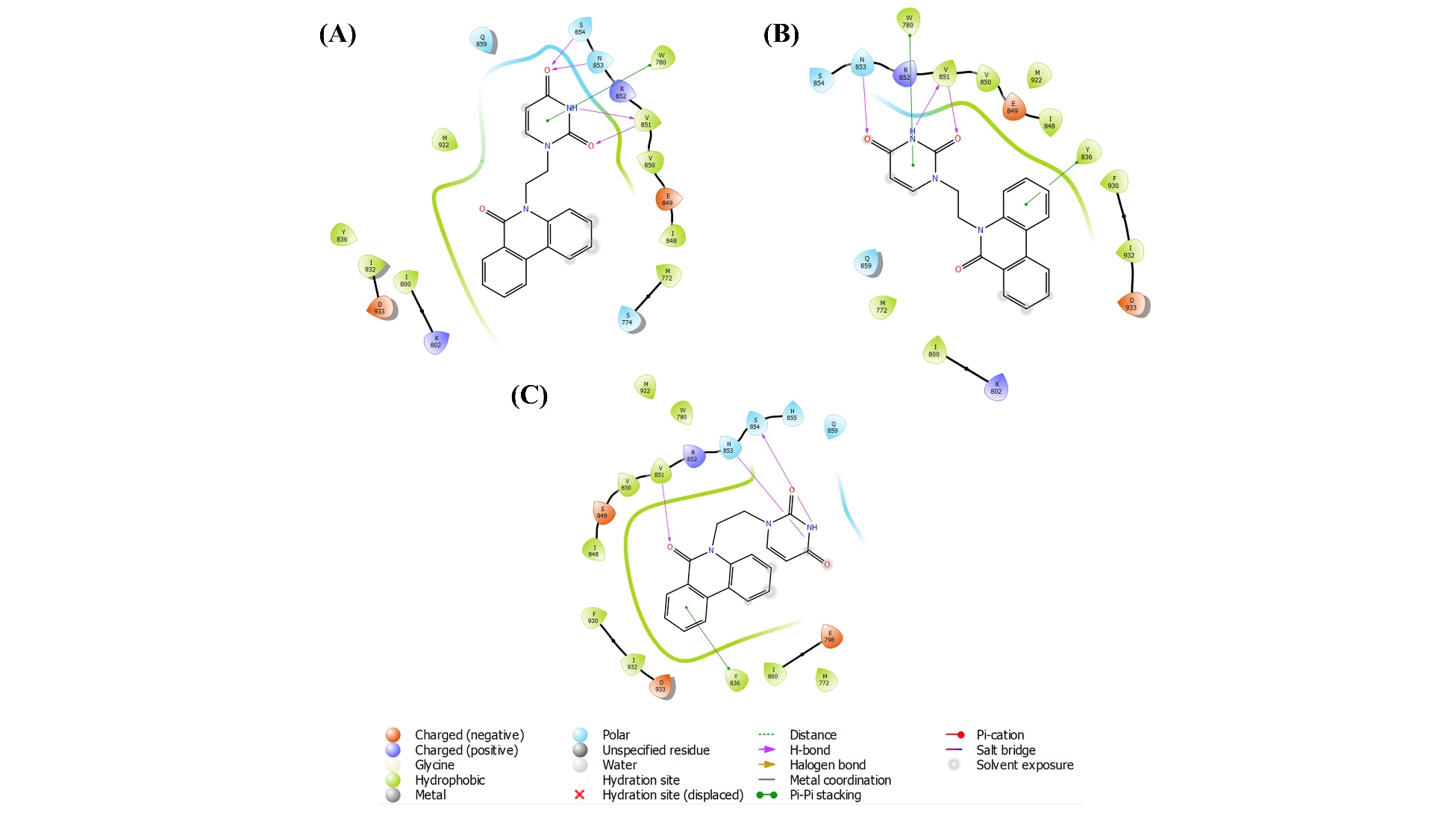


**Supplementary Figure S13.** 2D interaction diagram (IFD of top 3 poses) of Djh2 and PI3Kα.

\\
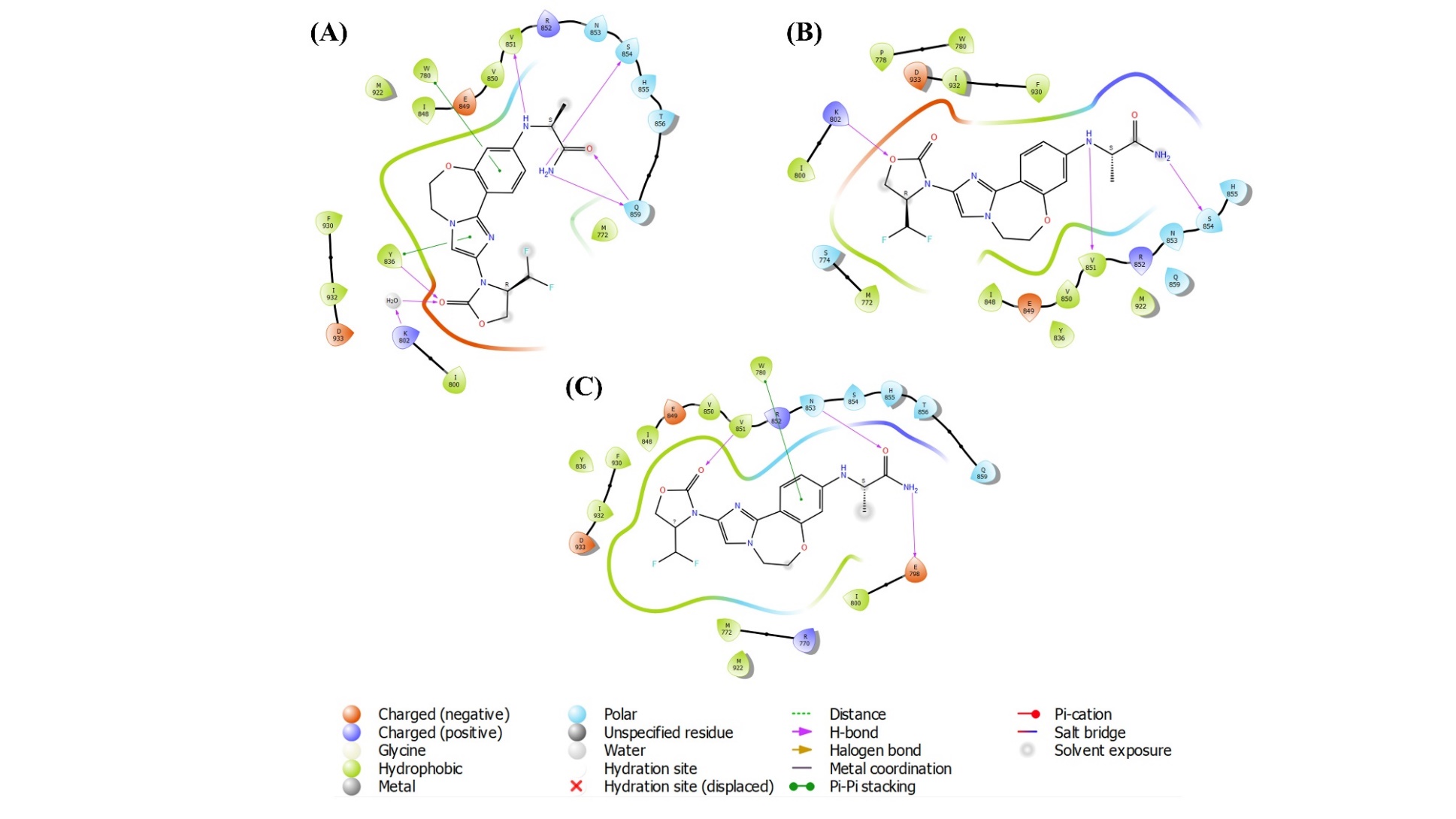


**Supplementary Figure S14.** 2D interaction diagram (IFD of top 3 poses) of Inavolisib and PI3Kα.


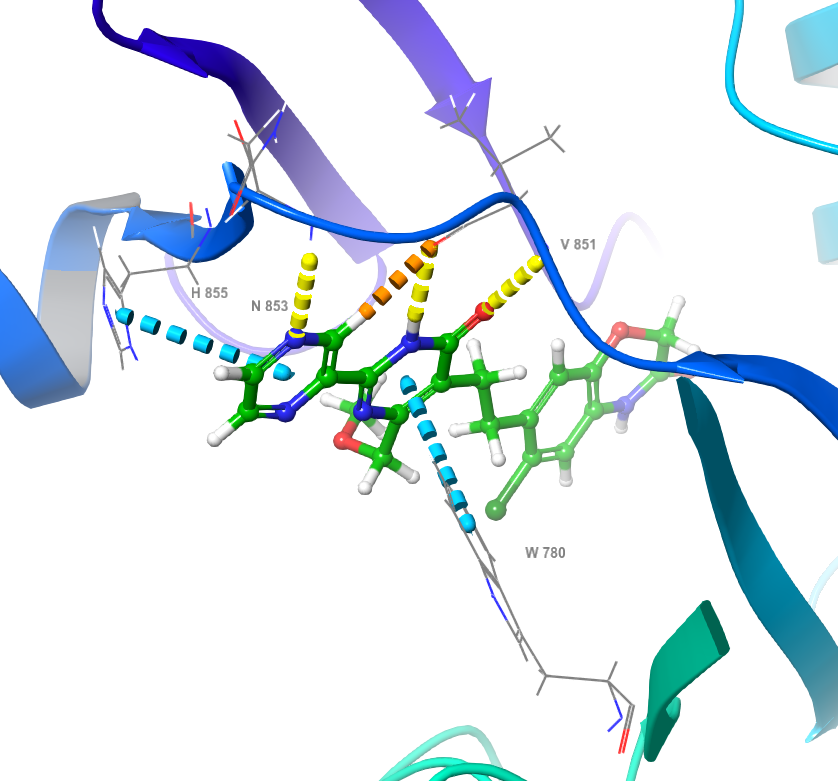


**Supplementary Figure S15.** 3D interaction diagram of Compound 10 and PI3Kα.


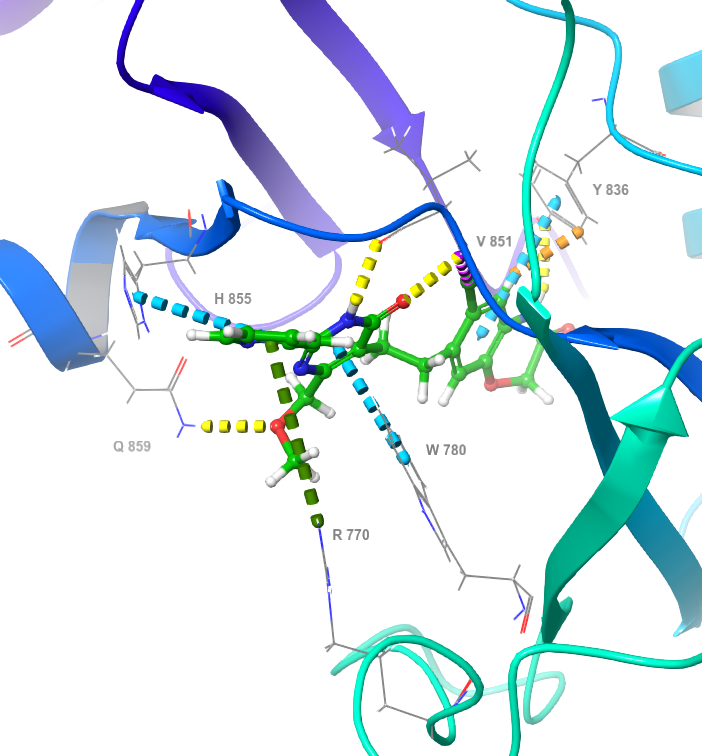


**Supplementary Figure S16.** 3D interaction diagram of Compound 06 and PI3Kα.


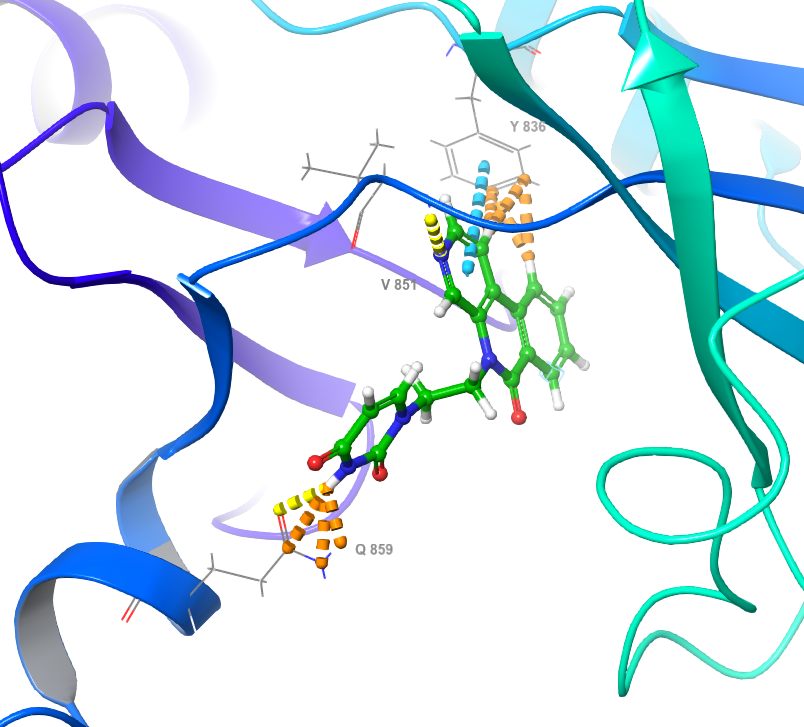


**Supplementary Figure S17.** 3D interaction diagram of Compound 08 and PI3Kα.


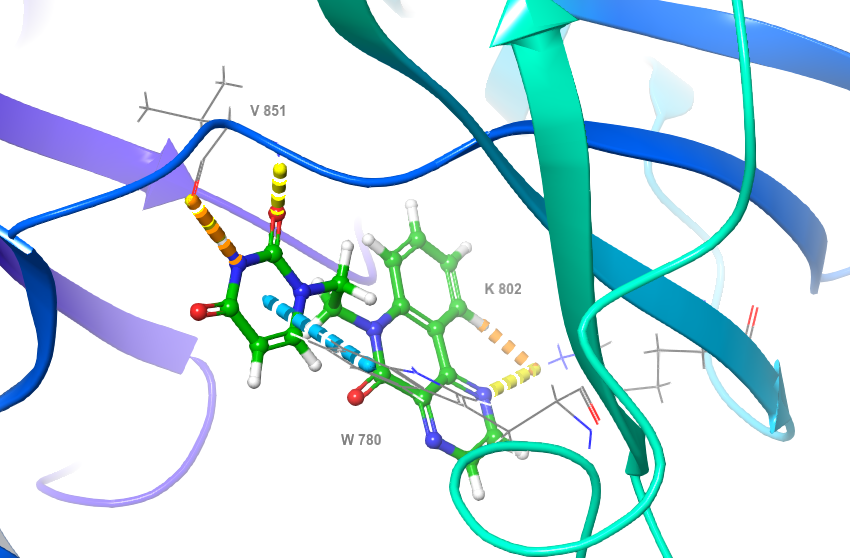


**Supplementary Figure S18.** 3D interaction diagram of Compound 19 and PI3Kα.


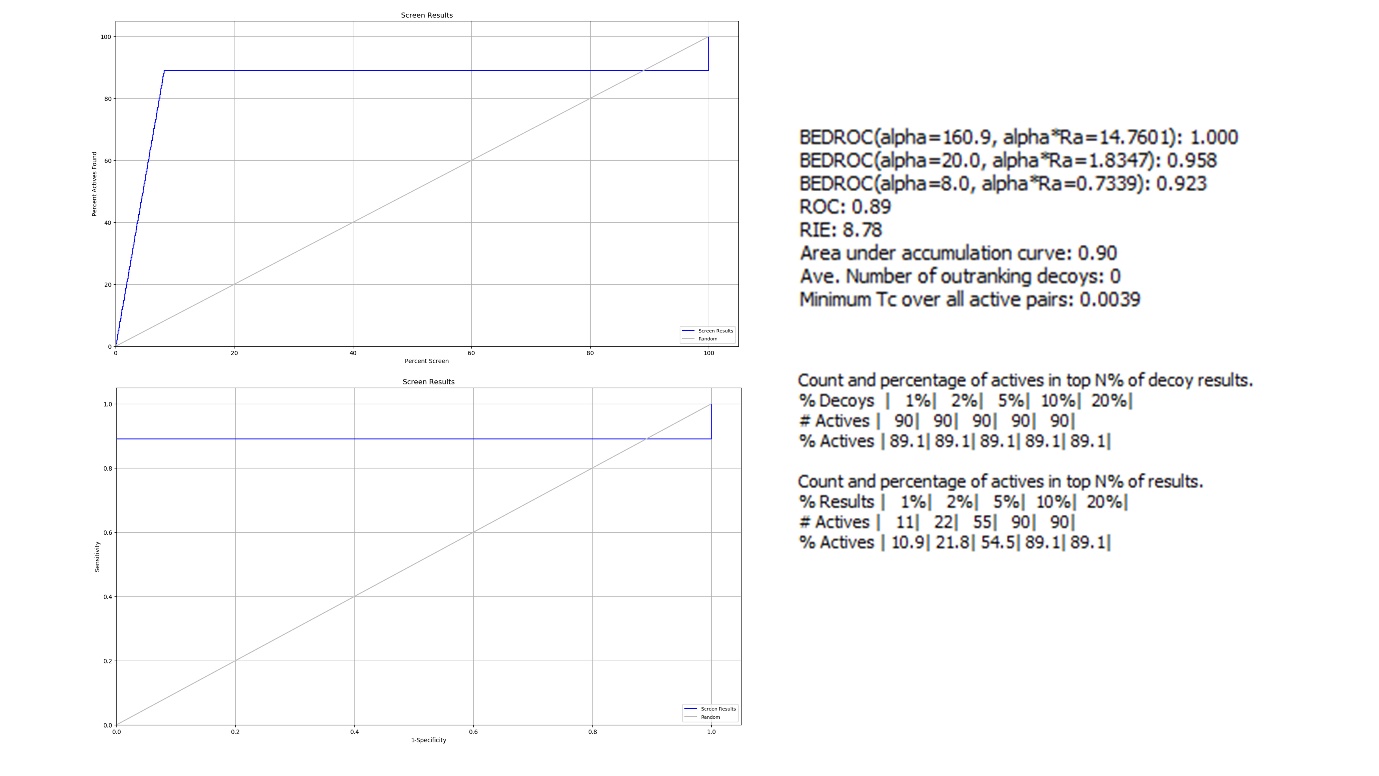


**Supplementary Figure S19.** Enrichment analysis of the molecular docking analysis of all the novel designed compounds by fragment based design on PI3Kα.


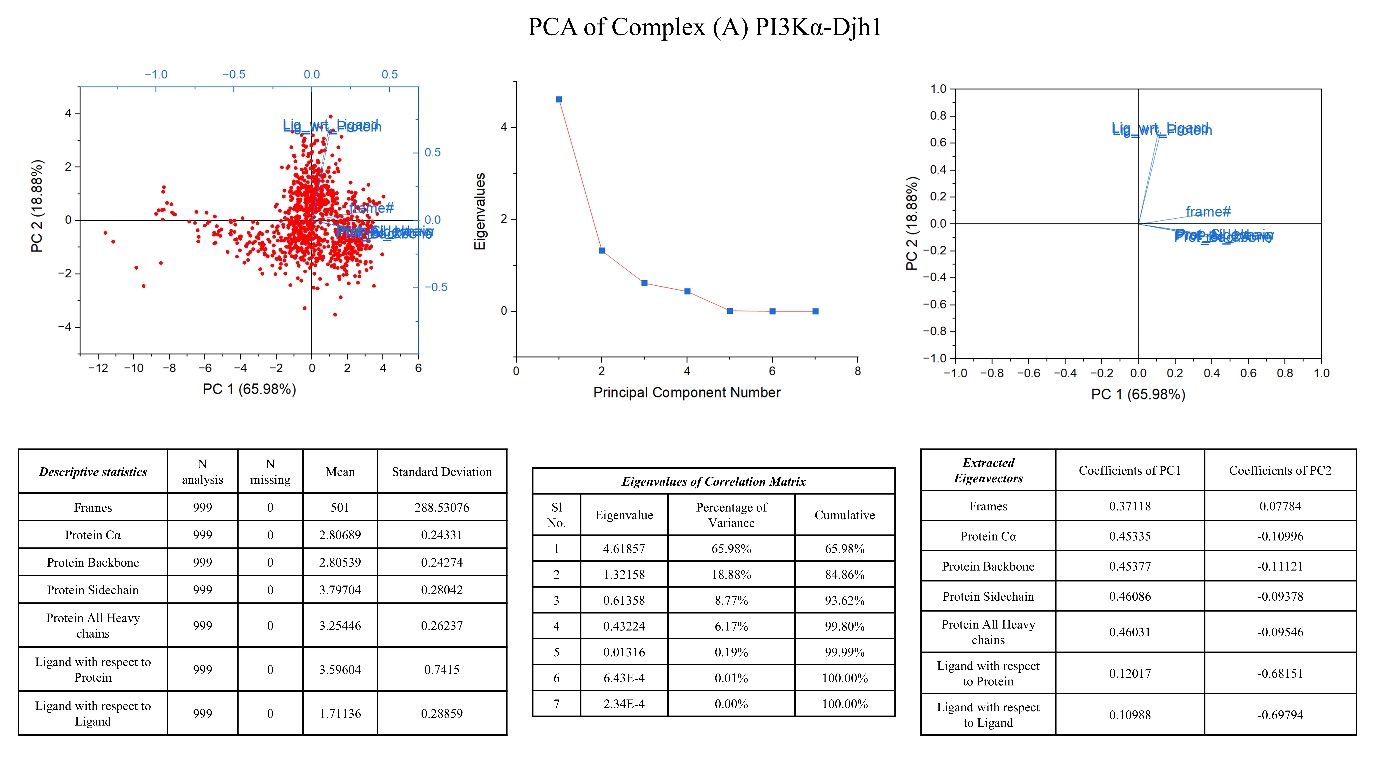


**Supplementary Figure S20.** Principle component analysis (PCA) of Complex (A) PI3Kα-Djh1.


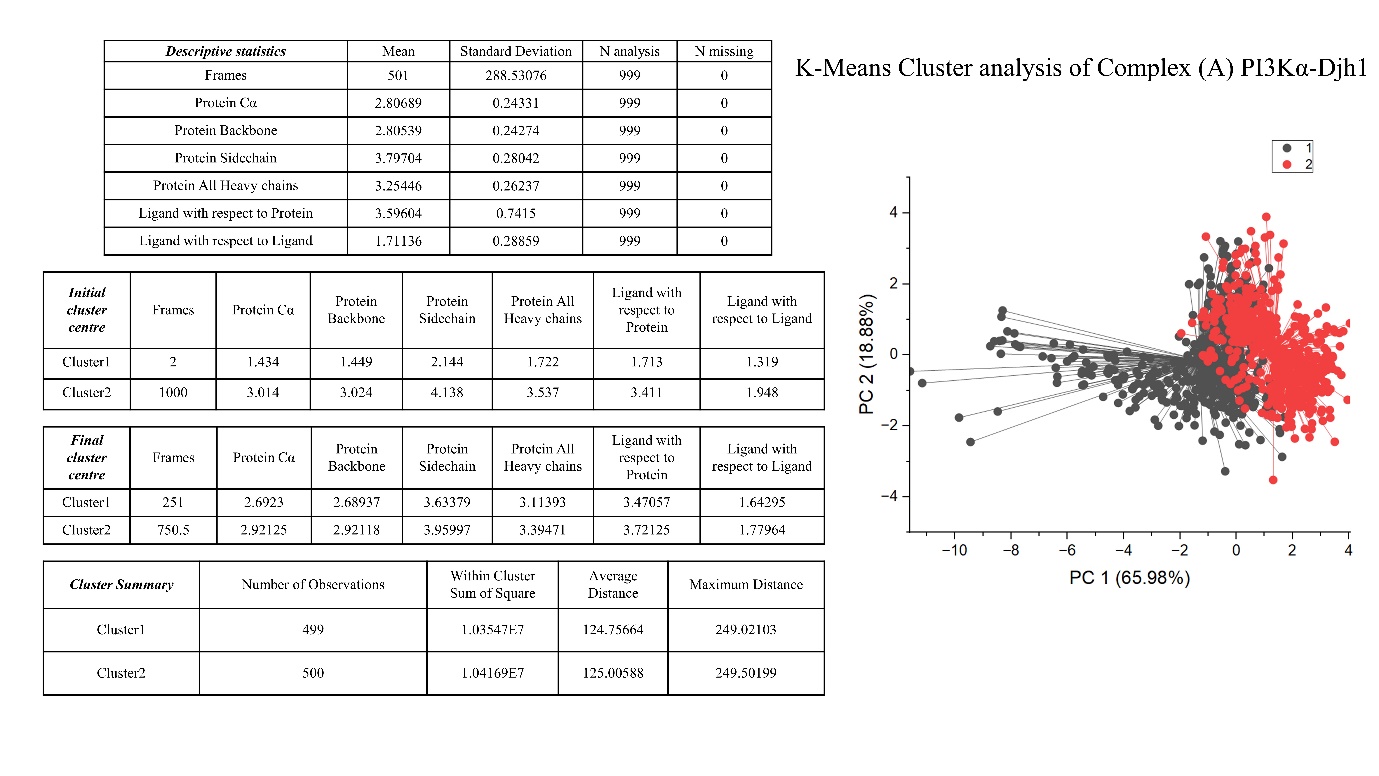


**Supplementary Figure S21.** K-Means cluster analysis of Complex (A) PI3Kα-Djh1.

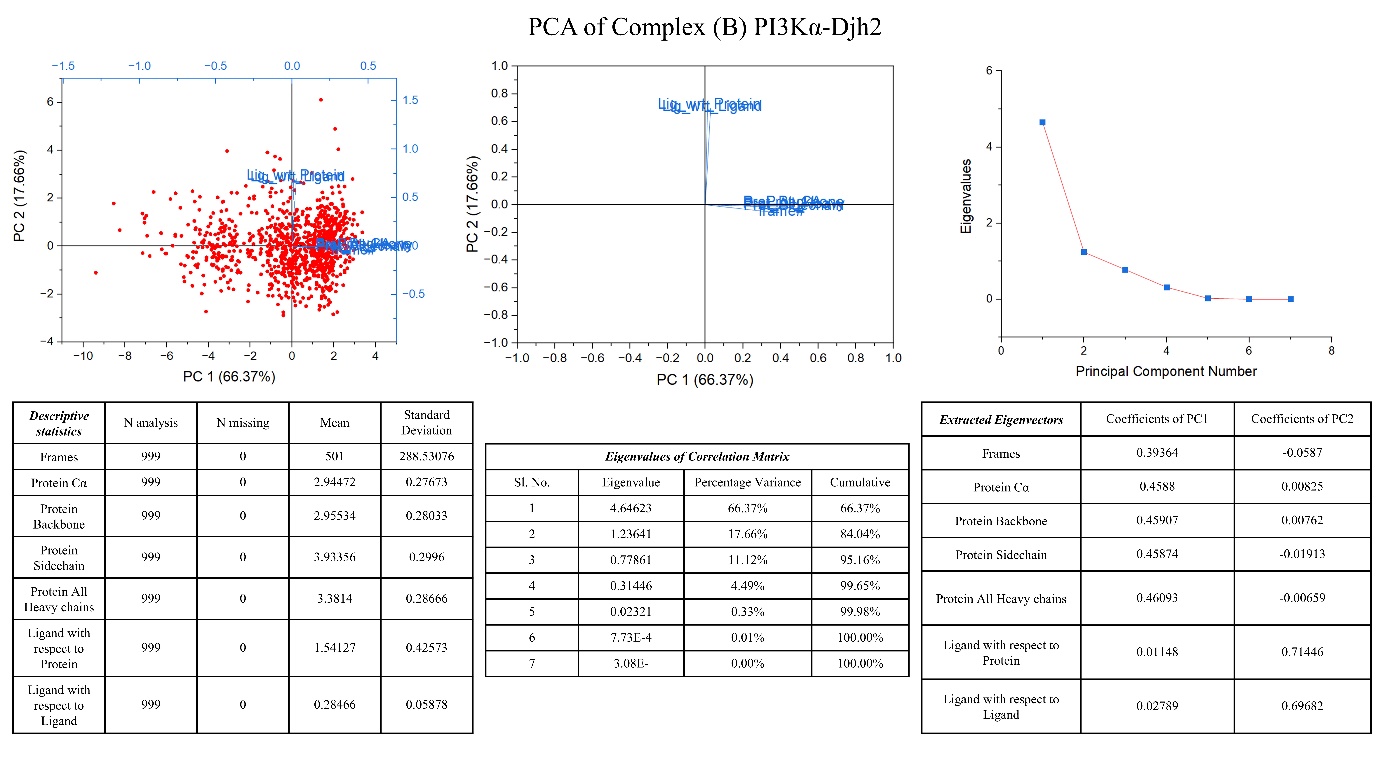


**Supplementary Figure S22.** Principle component analysis (PCA) of Complex (B) PI3Kα-Djh2.


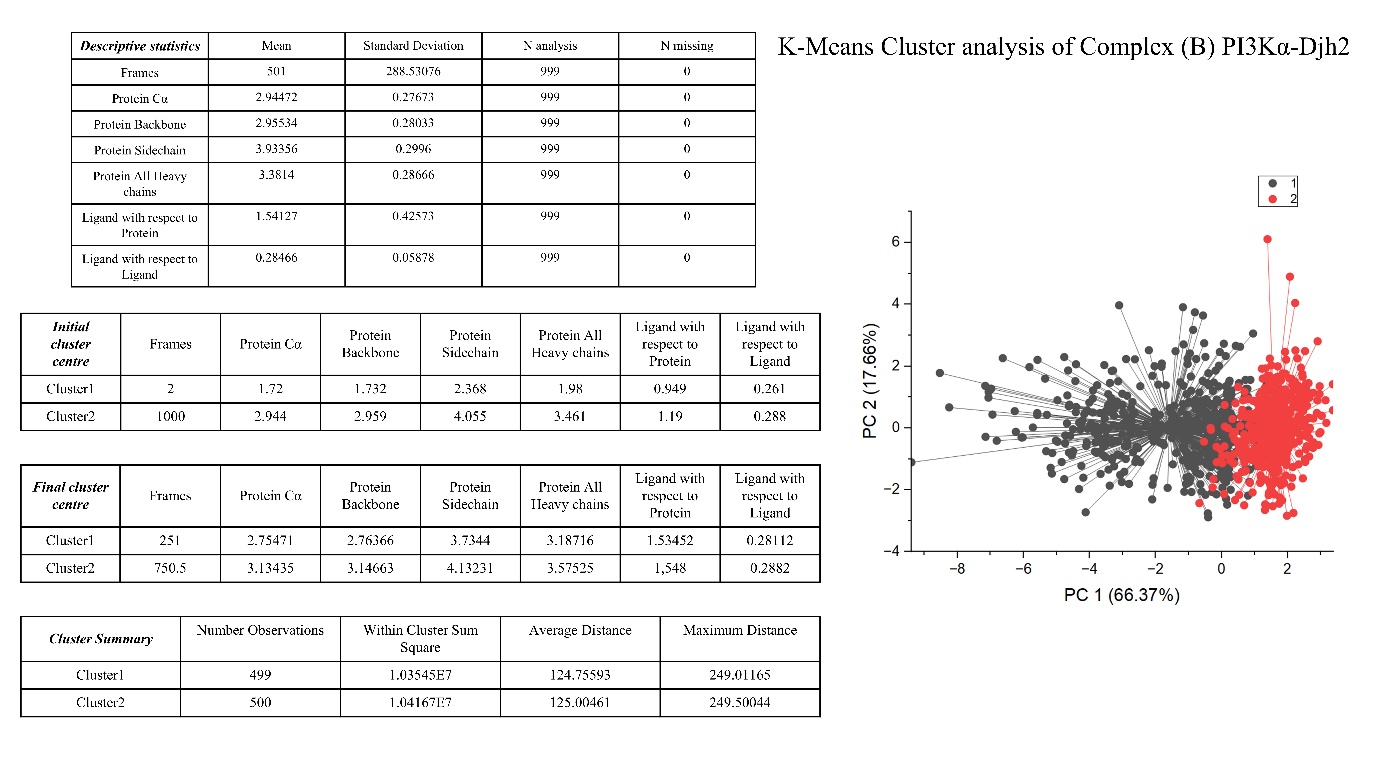


**Supplementary Figure S23.** K-Means cluster analysis of Complex (B) PI3Kα-Djh2.


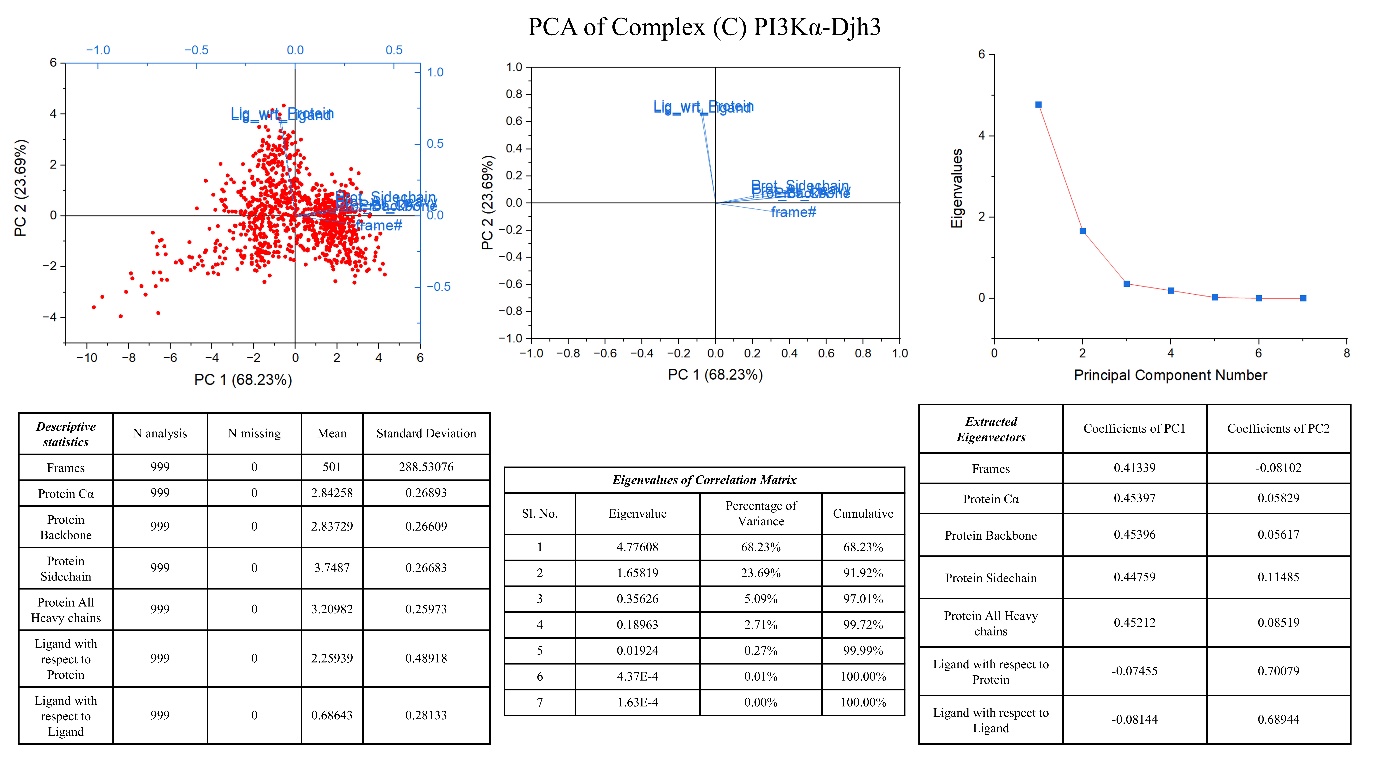


**Supplementary Figure S24.** Principle component analysis (PCA) of Complex (C) PI3Kα-Djh3.


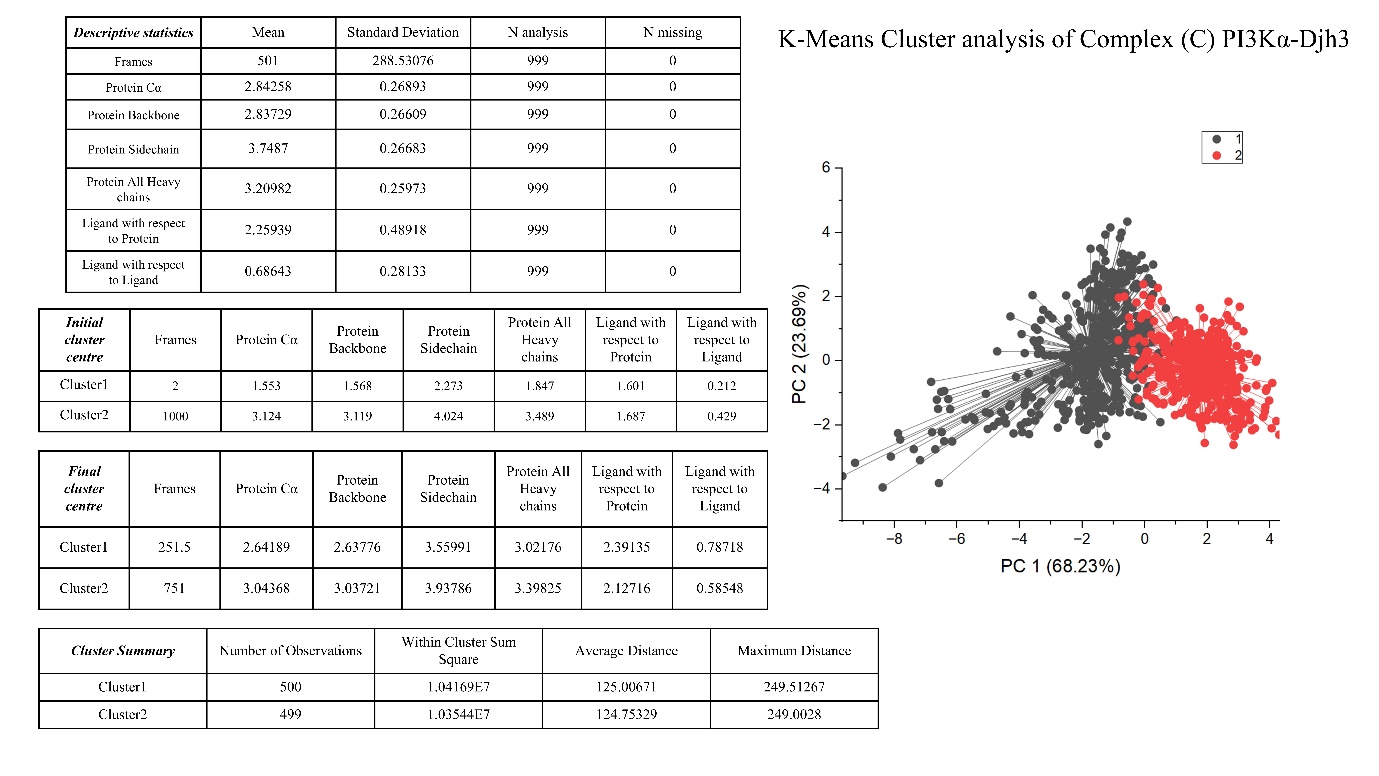


**Supplementary Figure S25.** K-Means cluster analysis of Complex (C) PI3Kα-Djh3.

**Supplementary Table**

**Supplementary Table 1.** Structure and IUPAC name of the top 10 compounds along wh standard Inavolisib.

| **Compound ID** | **Structure** | **IUPAC Name** |
| --- | --- | --- |
| Djh1 |  | 6-chloro-7-(2-(4-(methoxymethyl)-6-oxo-2-(pyridin-4-yl)-1,6-dihydropyrimidin-5-yl)ethyl)-2H-benzo[b][1,4]oxazin-3(4H)-one |
| Djh2 |  | 1-(2-(6-oxophenanthridin-5(6H)-yl)ethyl)pyrimidine-2,4(1H,3H)-dione |
| Djh3 |  | 7-(2-(2-amino-4-(3,4-dihydro-2H-benzo[b][1,4]dioxepin-7-yl)thiazol-5-yl)ethyl)-6-chloro-2H-benzo[b][1,4]oxazin-3(4H)-one |
| Djh4 |  | 8-(2-(6-amino-2-(4-aminophenyl)benzo[d]thiazol-4-yl)ethyl)-6-chloro-2H-benzo[b][1,4]oxazin-3(4H)-one |
| Djh5 |  | 7-(2-(2-amino-6-oxo-4-propyl-1,6-dihydropyrimidin-5-yl)ethyl)-6-chloro-2H-benzo[b][1,4]oxazin-3(4H)-one |
| Djh6 |  | 2-(5-(2-(6-chloro-3-oxo-3,4-dihydro-2H-benzo[b][1,4]oxazin-7-yl)ethyl)-2-(2-fluorophenyl)thiazol-4-yl)acetate |
| Djh7 |  | 6-chloro-7-(5-chloro-2-methoxy-3-(5,6,7,8-tetrahydro-[1,2,4]triazolo[4,3-a]pyrazine-7-carbonyl)phenethyl)-2H-benzo[b][1,4]oxazin-3(4H)-one |
| Djh8 |  | 2-(5-(2-(6-chloro-3-oxo-3,4-dihydro-2H-benzo[b][1,4]oxazin-7-yl)ethyl)-2-phenylthiazol-4-yl)acetate |
| Djh9 |  | 1-(5-((4-methyl-6-oxo-1,6-dihydropyrimidin-2-yl)amino)-2-(piperidin-1-yl)phenethyl)-5,6,7,8-tetrahydrobenzo[4,5]thieno[2,3-d]pyrimidine-2,4(1H,3H)-dione |
| Djh10 |  | 7-(2-(6-amino-9-(2-chloroethyl)-9H-purin-8-yl)ethyl)-6-chloro-2H-benzo[b][1,4]oxazin-3(4H)-one |
| Inavolisib |  | (2S)-2-[[2-[(4S)-4-(difluoromethyl)-2-oxo-1,3-oxazolidin-3-yl]-5,6-dihydroimidazo[1,2-d][1,4]benzoxazepin-9-yl]amino]propanamide |
| Alpelisib |  | [(2S)-N1-[4-methyl-5-[2-(1,1,1-trifluoro-2-methylpropan-2-yl)-4-pyridinyl]-2-thiazolyl]pyrrolidine-1,2-dicarboxamide](https://pubchem.ncbi.nlm.nih.gov/compound/%282S%29-N1-%5B4-methyl-5-%5B2-%281%2C1%2C1-trifluoro-2-methylpropan-2-yl%29-4-pyridinyl%5D-2-thiazolyl%5Dpyrrolidine-1%2C2-dicarboxamide) |
| Copanlisib |  | 2-amino-N-[7-methoxy-8-(3-morpholin-4-ylpropoxy)-2,3-dihydro-1H-imidazo[1,2-c]quinazolin-5-ylidene]pyrimidine-5-carboxamide |

**Supplementary Table S2.** 2D interaction diagram of molecular docking analysis of compounds – Djh3 to Djh10.

| **Compounds** | **2D interaction diagram** | **Interactions** |
| --- | --- | --- |
| Djh3 | 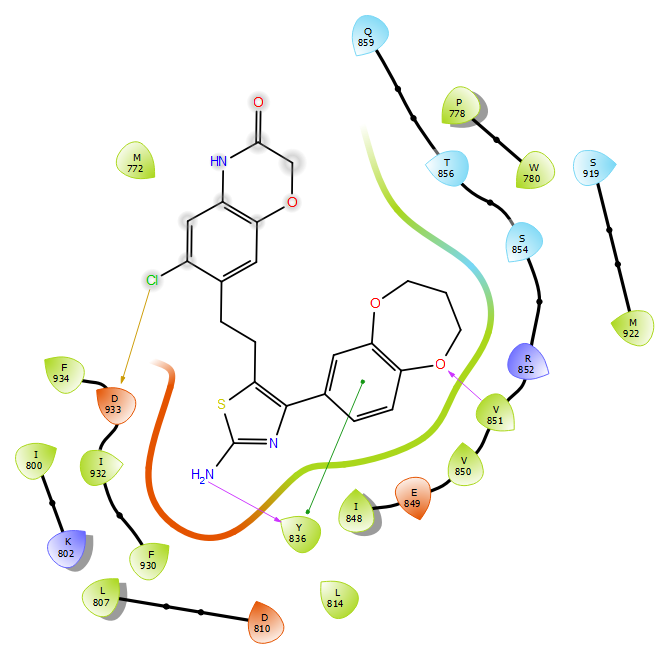 | **H-bond interactions:** V851, Y836, **π-π stacking:** Y836, **Halogen bond:** D933. |
| Djh4 | 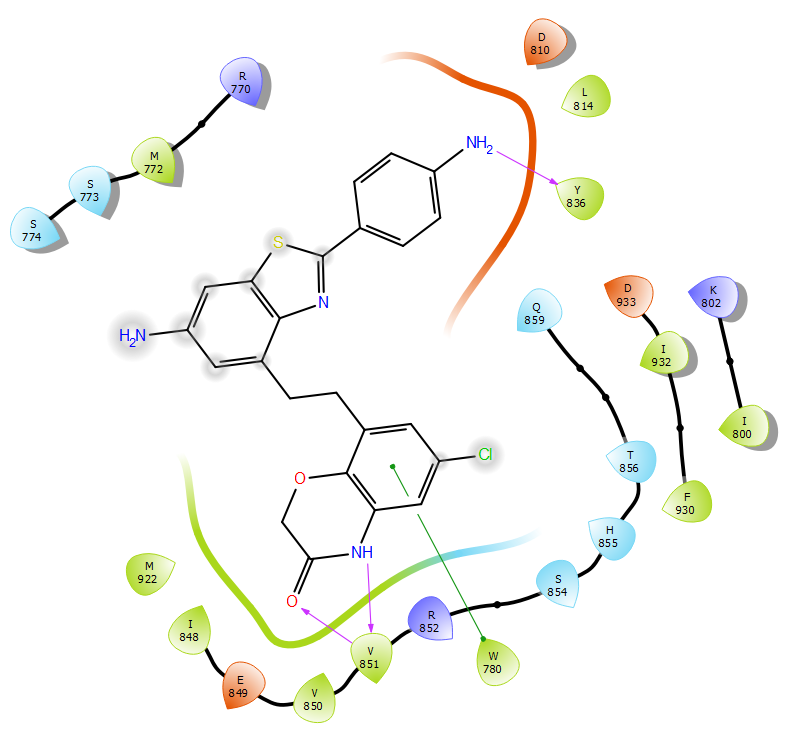 | **H-bond interactions:** V851, Y836, **π-π stacking:** W780. |
| Djh5 | 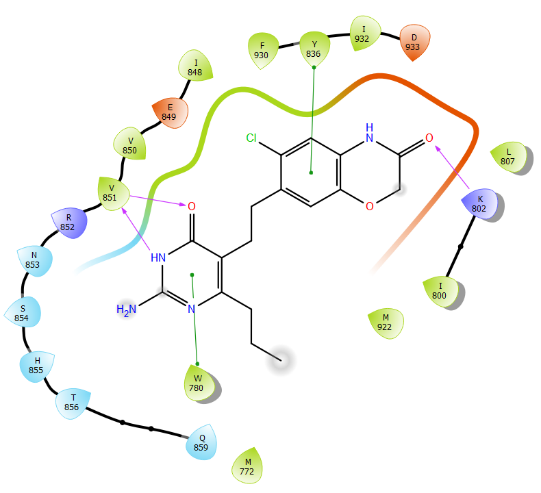 | **H-bond interactions:** V851, K802, **π-π stacking:** W780, Y836. |
| Djh6 | 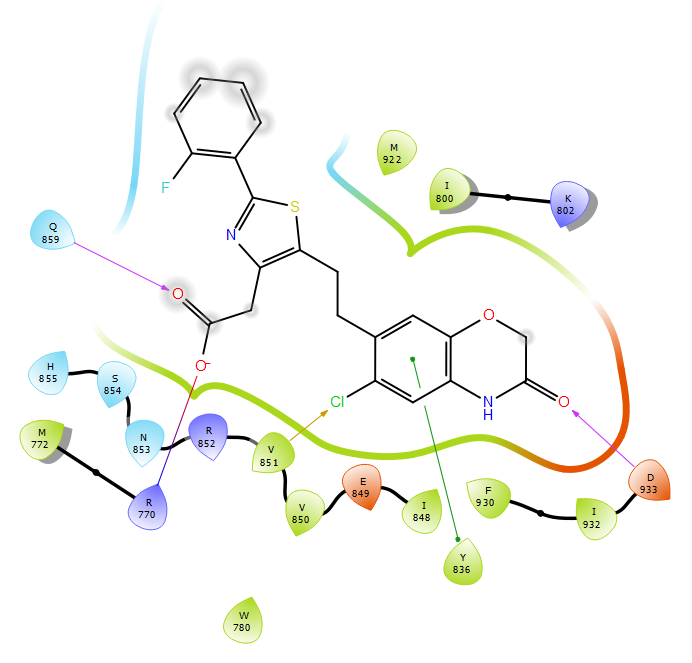 | **H-bond interactions:** Q859, D923, **π-π stacking:** Y836, **Halogen:** V851, **π-cation:** R770. |
| Djh7 | 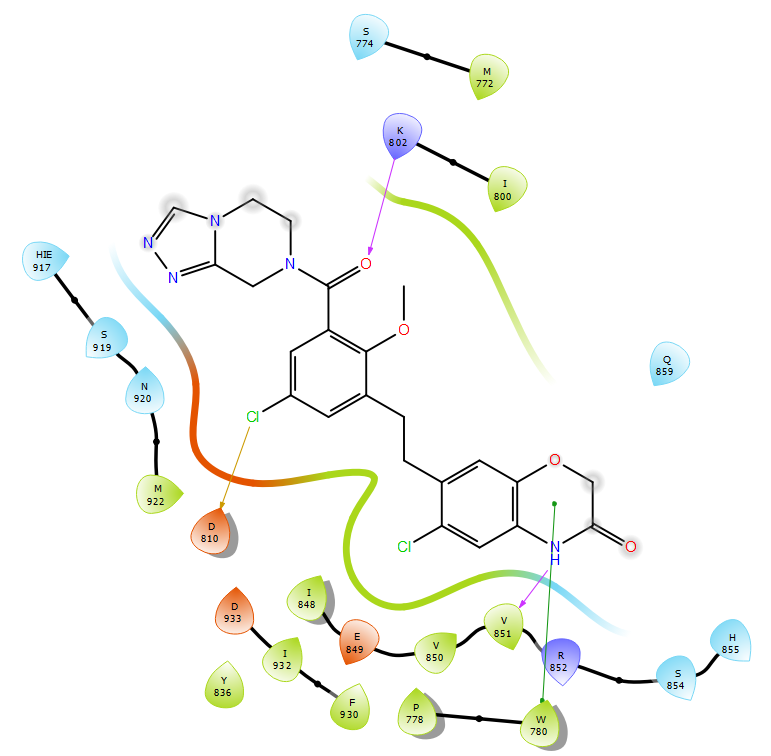 | **H-bond interactions:** V851, K802, **π-π stacking:** W780, **Halogen:** D810. |
| Djh8 | 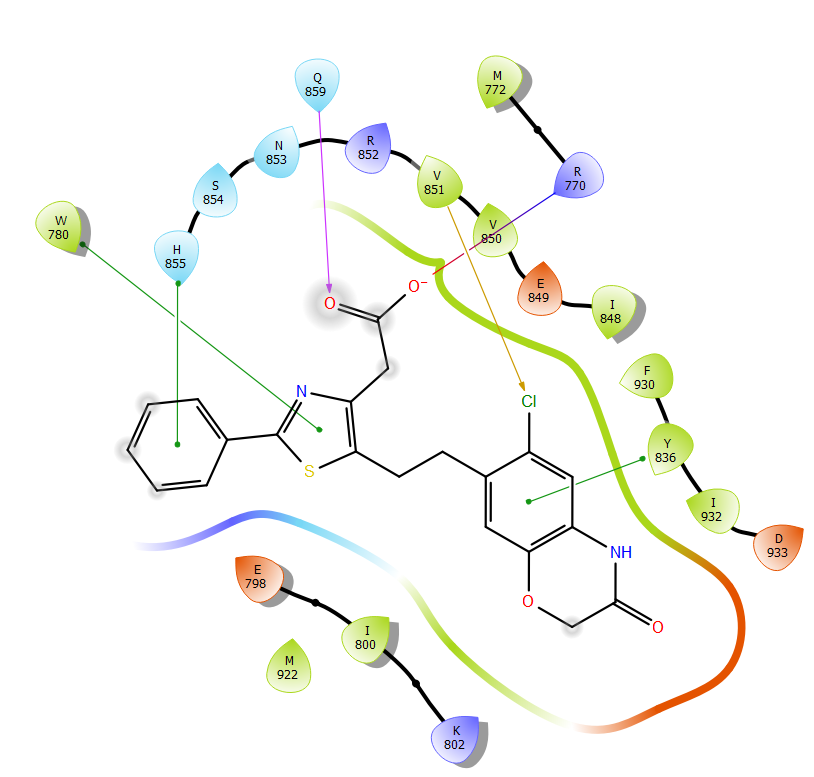 | **H-bond interactions:** Q859, **π-π stacking:** W780, Y836, H855, **Halogen:** V851,  **π -cation:** R770. |
| Djh9 | 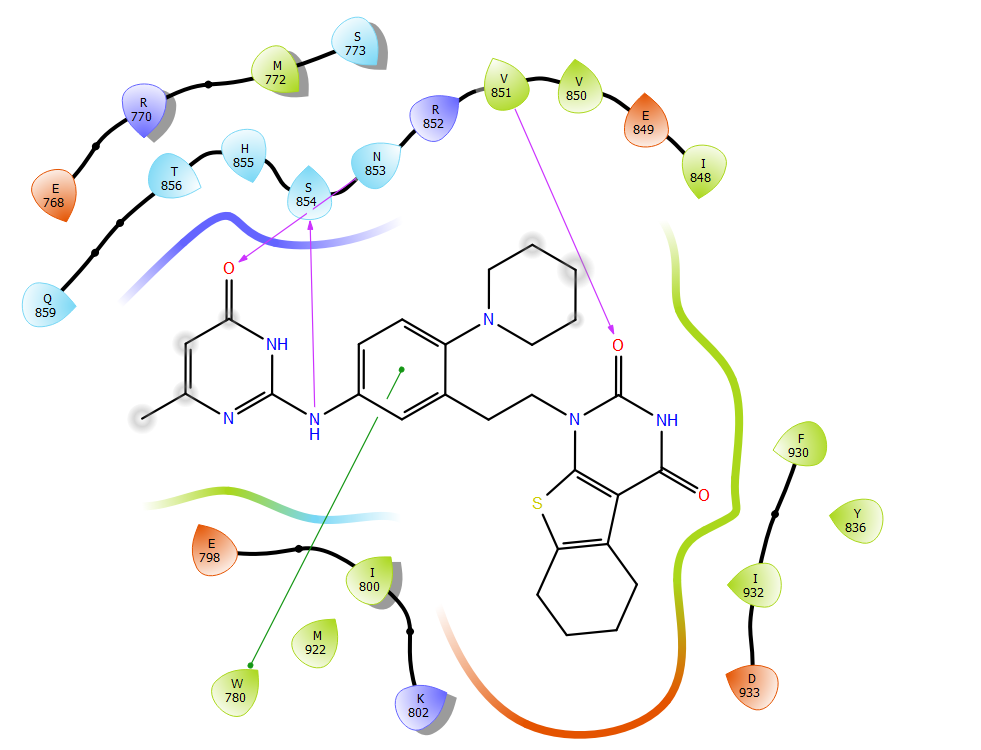 | **H-bond interactions:** V851, S854, N853, **π-π stacking:** W780. |
| Djh10 | 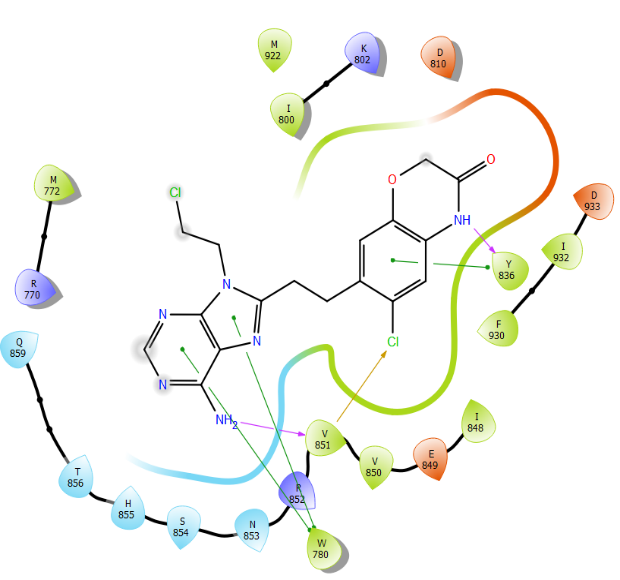 | **H-bond interactions:** V851, Y836, **π-π stacking:** W780, Y836,  **Halogen:** V851. |
| 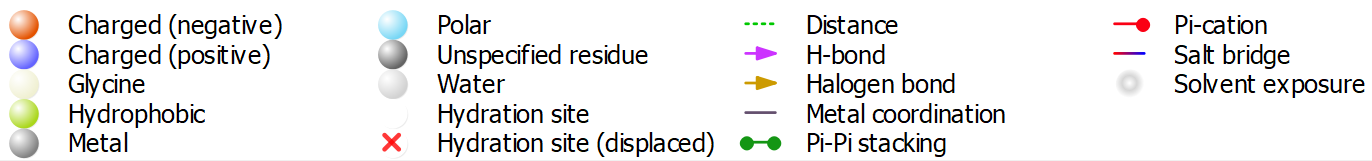  **[Note:** A (Alanine), R (Arginine), N (Asparagine), D (Aspartic acid), C (Cysteine), E (Glutamic acid), Q (Glutamine), G (Glycine), H (Histidine), I (Isoleucine), L (Leucine), K (Lysine), M (Methionine), F (Phenylalanine), P (Proline), S (Serine), T (Threonine), W (Tryptophan), Y (Tyrosine), V (Valine)**]** | | |

**Supplementary Table S3.** ADME analysis by pkCSM of compounds – Djh1 and Djh2, along with their bioisosteres.

| **Compound** | **Absorption** | | | | | | | **Distribution** | | | | **Metabolism** | | | | | | | **Excretion** | |
| --- | --- | --- | --- | --- | --- | --- | --- | --- | --- | --- | --- | --- | --- | --- | --- | --- | --- | --- | --- | --- |
|  | **Water**  **Solubility**  **(log mol/L)** | **Caco2 permeability**  **(log Papp in 10^-6^ cm/s)** | **Intestinal**  **Absorption**  **(human) (% absorbed)** | **Skin**  **Permeability (log Kp)** | **P- glycoprotein substrate (Yes/No)** | **P-glycoprotein I inhibitor (Yes/No)** | **P-glycoprotein II inhibitor (Yes/No)** | **VDss**  **(human) (log L/kg)** | **Fraction unbound (human) (Fu)** | **BBB permeability (log BB)** | **CNS permeability (log PS)** | **CYP2D6 substrate (Yes/No)** | **CYP3A4 substrate (Yes/No)** | **CYP1A2 inhibitor (Yes/No)** | **CYP2C19 inhibitor (Yes/No)** | **CYP2C9 inhibitor (Yes/No)** | **CYP 2D6 inhibitor (Yes/No)** | **CYP3A4 inhibitor (Yes/No)** | **Total Clearance (log ml/min/kg)** | **Renal OCT2 substrate (Yes/No)** |
| Djh1 | -3.949 | 0.783 | 87.962 | -2.738 | Yes | No | Yes | 0.272 | 0.029 | -1.272 | -3.549 | No | Yes | Yes | Yes | Yes | No | Yes | -0.069 | No |
| 10 | -3.793 | 0.86 | 78.903 | -2.736 | Yes | No | No | 0.219 | 0.029 | -1.385 | -1.385 | No | Yes | No | No | Yes | No | Yes | 0.1 | No |
| 06 | -3.667 | 0.61 | 87.844 | -2.742 | Yes | No | Yes | 0.292 | 0.079 | -1.289 | -3.533 | No | Yes | No | Yes | Yes | No | Yes | -0.074 | No |
| Dhj2 | -3.286 | 1.173 | 99.029 | -2.742 | Yes | No | Yes | -0.09 | 0.182 | -.03 | -2.412 | No | Yes | Yes | No | No | No | No | 1.064 | No |
| 08 | -2.644 | 1.267 | 65.825 | -2.763 | Yes | No | No | 0.391 | 0.246 | -0.695 | -2.681 | No | Yes | Yes | No | No | No | Yes | 1.059 | No |
| 19 | -2.58 | 0.167 | 84.869 | -2.735 | No | No | No | 0.269 | 0.18 | -0.879 | -3.002 | No | Yes | Yes | No | No | No | Yes | 1.133 | No |
| Inavolisib | -2.927 | 0.992 | 84.483 | -2.738 | Yes | No | No | -0.73 | 0.193 | -0.575 | -2.972 | No | Yes | No | No | No | No | No | 0.617 | No |

**Supplementary Table S4.** The docking scores of Djh1 to Djh10, with their mean and standard error of mean (SEM).

| **Compounds** | **XP docking scores**  **(Kcal/mol)** | **SP docking scores (Kcal/mol)** | **Mean**  **Docking scores (Kcal/mol)** | **SEM** |
| --- | --- | --- | --- | --- |
| Djh1 | -10.214 | -9.202 | -9.708 | 0.506 |
| Djh2 | -10.126 | -8.774 | -9.45 | 0.676 |
| Djh3 | -10.122 | -8.526 | -9.324 | 0.798 |
| Djh4 | -9.308 | -8.518 | -8.913 | 0.395 |
| Djh5 | -9.127 | -8.42 | -8.7735 | 0.3535 |
| Djh6 | -9.04 | -8.172 | -8.606 | 0.434 |
| Djh7 | -8.63 | -7.917 | -8.2735 | 0.3565 |
| Djh8 | -8.172 | -7.812 | -7.992 | 0.18 |
| Djh9 | -8.144 | -7.662 | -7.903 | 0.241 |
| Djh10 | -8.139 | -7.599 | -7.869 | 0.27 |
| Inavolisib | -5.922 | -5.36 | -5.641 | 0.281 |
| Alpelisib | -9.295 | -7.101 | -8.198 | 1.097 |
| Copanlisib | -4.029 | -3.853 | -3.941 | 0.088 |

**Supplementary Table S5.** The MMGBSA and SASA scores of the top ten compounds along with standard error of mean (SEM) analysis.

| **Compounds** | **MMGBSA (ΔG) XP** | **MMGBSA (ΔG) SP** | **Mean MMGBSA (ΔG)** | **SEM** | **MMGBSA (ΔG-Coulomb) XP** | **MMGBSA (ΔG-Coulomb) SP** | **Mean MMGBSA (ΔG-Coulomb)** | **SEM** | **SASA** |
| --- | --- | --- | --- | --- | --- | --- | --- | --- | --- |
| Djh1 | -60.7 | -51.74 | -56.22 | 4.48 | -12.92 | -11.21 | -12.065 | 0.855 | 478.168 |
| Djh2 | -45.64 | -32.57 | -39.105 | 6.535 | -5.6 | -5 | -5.3 | 0.3 | 408.104 |
| Djh3 | -43.2 | -30.28 | -36.74 | 6.46 | -12.97 | -8.53 | -10.75 | 2.22 | 436.787 |
| Djh4 | -58.36 | -39.25 | -48.805 | 9.555 | -16.25 | -12.52 | -14.385 | 1.865 | 354.017 |
| Djh5 | -54.32 | -43.58 | -48.95 | 5.37 | -15.86 | -11.57 | -13.715 | 2.145 | 377.26 |
| Djh6 | -63.18 | -35.53 | -49.355 | 13.825 | -5.02 | -2.52 | -3.77 | 1.25 | 451.66 |
| Djh7 | -63.04 | -47.52 | -55.28 | 7.76 | -11.42 | -5.68 | -8.55 | 2.87 | 354.017 |
| Djh8 | -67.98 | -51.85 | -59.915 | 8.065 | -4.61 | -3.58 | -4.095 | 0.515 | 446.08 |
| Djh9 | -60.71 | -41.3 | -51.005 | 9.705 | -19.57 | -15.7 | -17.635 | 1.935 | 393.844 |
| Djh10 | -64.2 | -50.2 | -57.2 | 7 | -4.54 | -4.28 | -4.41 | 0.13 | 354.017 |
| Inavolisib | -57.51 | -46.52 | -52.015 | 5.495 | -20.54 | -16.34 | -18.44 | 2.1 | 635.095 |
| Alpelisib | -50.27 | -41.55 | -45.91 | 4.36 | -12.78 | -9.14 | -10.96 | 1.82 | 711.630 |
| Copanlisib | -21.56 | -7.58 | -14.57 | 6.99 | -3.55 | -1.08 | -2.315 | 1.235 | ND |
| ND = Cannot be determined using Qikprop tool of Maestro, Schrodinger. | | | | | | | | | |
